# Aging impairs the essential contributions of non‐glial progenitors to neurorepair in the dorsal telencephalon of the Killifish *Nothobranchius furzeri*

**DOI:** 10.1111/acel.13464

**Published:** 2021-08-24

**Authors:** Jolien Van houcke, Valerie Mariën, Caroline Zandecki, Sophie Vanhunsel, Lieve Moons, Rajagopal Ayana, Eve Seuntjens, Lutgarde Arckens

**Affiliations:** ^1^ Department of Biology Laboratory of Neuroplasticity and Neuroproteomics KU Leuven Leuven Belgium; ^2^ Department of Biology Laboratory of Developmental Neurobiology KU Leuven Leuven Belgium; ^3^ Department of Biology Laboratory of Neural Circuit Development and Regeneration KU Leuven Leuven Belgium; ^4^ KU Leuven Brain Institute Leuven Belgium

**Keywords:** aging, glial scar, inflammatory response, Killifish, neurodegenerative diseases, neuroregeneration, teleost, traumatic brain injury

## Abstract

The aging central nervous system (CNS) of mammals displays progressive limited regenerative abilities. Recovery after loss of neurons is extremely restricted in the aged brain. Many research models fall short in recapitulating mammalian aging hallmarks or have an impractically long lifespan. We established a traumatic brain injury model in the African turquoise killifish (*Nothobranchius furzeri*), a regeneration‐competent vertebrate that evolved to naturally age extremely fast. Stab‐wound injury of the aged killifish dorsal telencephalon unveils an impaired and incomplete regeneration response when compared to young individuals. In the young adult killifish, brain regeneration is mainly supported by atypical non‐glial progenitors, yet their proliferation capacity clearly declines with age. We identified a high inflammatory response and glial scarring to also underlie the hampered generation of new neurons in aged fish. These primary results will pave the way to unravel the factor age in relation to neurorepair, and to improve therapeutic strategies to restore the injured and/or diseased aged mammalian CNS.

## Graphical abstract

Neuroregeneration after stab‐wound injury is incomplete in aged killifish, caused by reduced proliferation of specialized non‐glial progenitors (NGPs) and mammalian‐like glial scarring. It seems that aged killifish deploy the restricted regenerative program, typically associated with mammals, instead of successful neurorepair as shown here for young adult killifish.

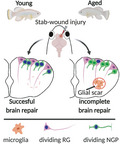

**Abbreviations**
AMC: age‐matched control
BBB: blood‐brain barrier
BLBP: brain lipid‐binding protein
CNS: central nervous system
Csf1ra: colony‐stimulating factor 1 receptor a
D: dorsal
Dl: dorso‐lateral
Dp: dorso‐posterior
Dpi: days post injury
GFAP: glial fibrillary acidic protein
GS: glutamine synthetase
hpi: hours post injury
IL8: interleukin 8
nGen: newly generated
NGP: non‐glial progenitor
NPC: neuronal progenitor cell
NSC: neural stem cell
OD: optical density
POL: polarized light
PVZ: periventricular zone
RG: radial glia
RMS: rostral migratory stream
SA β‐gal: senescence‐associated β‐galactosidase
SASP: senescence‐associated secretory phenotype
SGZ: subgranular zone
SR: picro sirius red
SVZ: subventricular zone
TBI: traumatic brain injury
VZ: ventricular zone
WFA: wisteria floribunda agglutinin

## 1 INTRODUCTION

Age‐related neurodegenerative diseases are highly debilitating and incurable pathologies that impinge a high socio‐economic burden on our society (El‐Hayek et al., 2019). They share a progressive degeneration of neurons, which results in loss of brain function and a heterogeneous array of incapacitating symptoms (Dugger & Dickson, 2017). Therapeutic strategies for brain restoration consist of compensating for neuronal loss by generating new neurons from the existing stem cell pools that can integrate into the existing circuitry. The capacity for neuroregeneration is naturally limited in the adult mammalian brain (Zhao et al., 2016). Neural stem cells may start dividing upon injury, but a large part of the newly generated neurons fail to mature, survive, and integrate into the existing neural network, thereby restricting full recovery (Arvidsson et al., 2002; Grade & Götz, 2017; Turnley et al., 2014). The mammalian neurogenic potential diminishes even further with advancing age, which constitutes one of the main risk factors for neurodegenerative diseases (Galvan & Jin, 2007; Hou et al., 2019; Popa‐Wagner et al., 2011). Studying vertebrate aging in models with high neuroregenerative capacities, such as teleost fish, can therefore help reveal key information to deal with each of these physiological brakes on neuroregeneration and neurorepair.

Zebrafish and killifish share, respectively, 70% (Howe et al., 2013) and 60% (calculated via Ensembl BioMart) of their coding genes with humans, but in contrast to mammals, retain the ability to regenerate multiple organs, including fin, heart, and the central nervous system (CNS) (Marques et al., 2019; Zupanc & Sîrbulescu, 2012). They have been extensively exploited as gerontology models in the past (Van houcke et al., 2021). Yet, most teleosts are relatively long‐lived (3–5 years) just like mice, making investigations about the specific impact of age impractical (Van houcke et al., 2021). The African turquoise killifish (*Nothobranchius furzeri*), however, has surfaced as an ideal vertebrate model for aging studies because of its extremely short lifespan. The short‐lived GRZ strain for example, has a median lifespan of 4–6 months depending on housing conditions (Polačik et al., 2016; Valdesalici & Cellerino, 2003; Valenzano et al., 2015). Killifish naturally live in ephemeral ponds in Africa, which have forced this species to evolve into a short lifespan and rapid aging (Genade et al., 2005). Many typical aging hallmarks of mammals are conserved in killifish (Van houcke et al., 2021). Understanding how aged killifish retain or lose their high regenerative capacity upon CNS aging could thus catalyze the development of therapeutic strategies aiming at inducing successful neuroregeneration in the adult mammalian brain.

The telencephalon or forebrain of fish is a favorable model of study since homologues to the two main neurogenic zones of mammals, the subgranular zone (SGZ) of the hippocampus and the subventricular zone (SVZ) of the lateral ventricles, have been identified in the pallium and subpallium (Ghaddar et al., 2021). It was recently discovered that the killifish dorsal pallium holds two classes of progenitors: (1) the commonly known radial glia (RGs) and (2) the non‐glial progenitors (NGPs). NGPs are devoid of the typical astroglial/RG markers, such as glutamine synthetase (GS), brain lipid‐binding protein (BLBP), glial fibrillary acidic protein (GFAP), and vimentin, and have a morphology that is less branched than RGs (Coolen et al., 2020). Early in development, killifish RGs enter a premature quiescent state and proliferation is supported by the NGPs. This is in sharp contrast to the situation in zebrafish, where RGs represent the neurogenic population in the dorsal pallium (Coolen et al., 2020; Kroehne et al., 2011; Rothenaigner et al., 2011). How these two different progenitor classes, with the NGPs appearing unique to the killifish, behave in the neuroregenesis process, and whether their neurogenic capacity is influenced by aging, remains unexplored.

In the present study, we have first set up and validated stab‐wound injury as a reliable traumatic brain injury (TBI) model. Next, we have decoded the impact of aging on the regeneration capacity—and on the two neurogenic pools—in the killifish dorsal pallium after stab‐wound injury. We find that aged killifish, just like mammals, are not capable to successfully regenerate; they develop a high inflammatory reaction and glial scarring, show diminished injury‐induced neurogenesis, and the vast majority of newborn neurons fail to reach the injury site. Remarkably, we show neuroregeneration to be supported by the NGPs in both young adult and aged killifish, and not the RGs that typically constitute neuroregenesis in other teleosts. Taken together, aged killifish appear to mimic the impaired regeneration capacity also seen in adult and/or aged mammals, instead of displaying the high regenerative capacities seen in young adult teleost fish, including killfish. In summary, we propose the killifish aging nervous system as a valuable model to create knowledge about the identity and mode of action of drivers and brakes of neuroregenerative properties. This may eventually elucidate how to boost the repair capacity in an aging context in the diseased/injured mammalian brain.

## 2 RESULTS

### 2.1 A stab‐wound injury model to study neuroregeneration in the killifish pallium

Before studying neuroregeneration in the young and aged dorsal pallium of killifish, we first introduced an easy‐to‐use and reproducible brain injury model. Stab‐wound injuries have been extensively characterized in the zebrafish telencephalon and effectively induce brain regeneration (Ghaddar et al., 2021). We chose to optimize a similar injury model in the young adult (6‐week‐old) and aged (18‐week‐old) killifish telencephalon (Figure S1A–B, Video S1). Approaching the dorsal telencephalon through the nostrils was not possible without damaging the large eyes, since the nostrils of the killifish are positioned more lateral compared to zebrafish. Instead, the medial part of the right telencephalic hemisphere of young and aged killifish was targeted from above, and a 33‐gauge Hamilton needle was pushed through the skull, approximately 500 µm in depth (Figure S1C, Video S1). The medial telencephalon lies in between the eyes, which were used as visual landmarks. Skin and fat tissue were removed to visualize the skull of the fish. Just prior to wounding, the needle was dipped in Vybrant DiD Cell‐Labeling Solution in order to permanently label the cells close to the injury site. This DiD dye approach enabled reconstruction of the original injury site, even if complete recovery had taken place, and the wounded area could no longer be distinguished from the surrounding tissue by (immuno)histology (Figure S1C–K, Video S1).

### 2.2 Aging impairs tissue recovery after stab‐wound injury by induction of a glial scar

**FIGURE 1:**
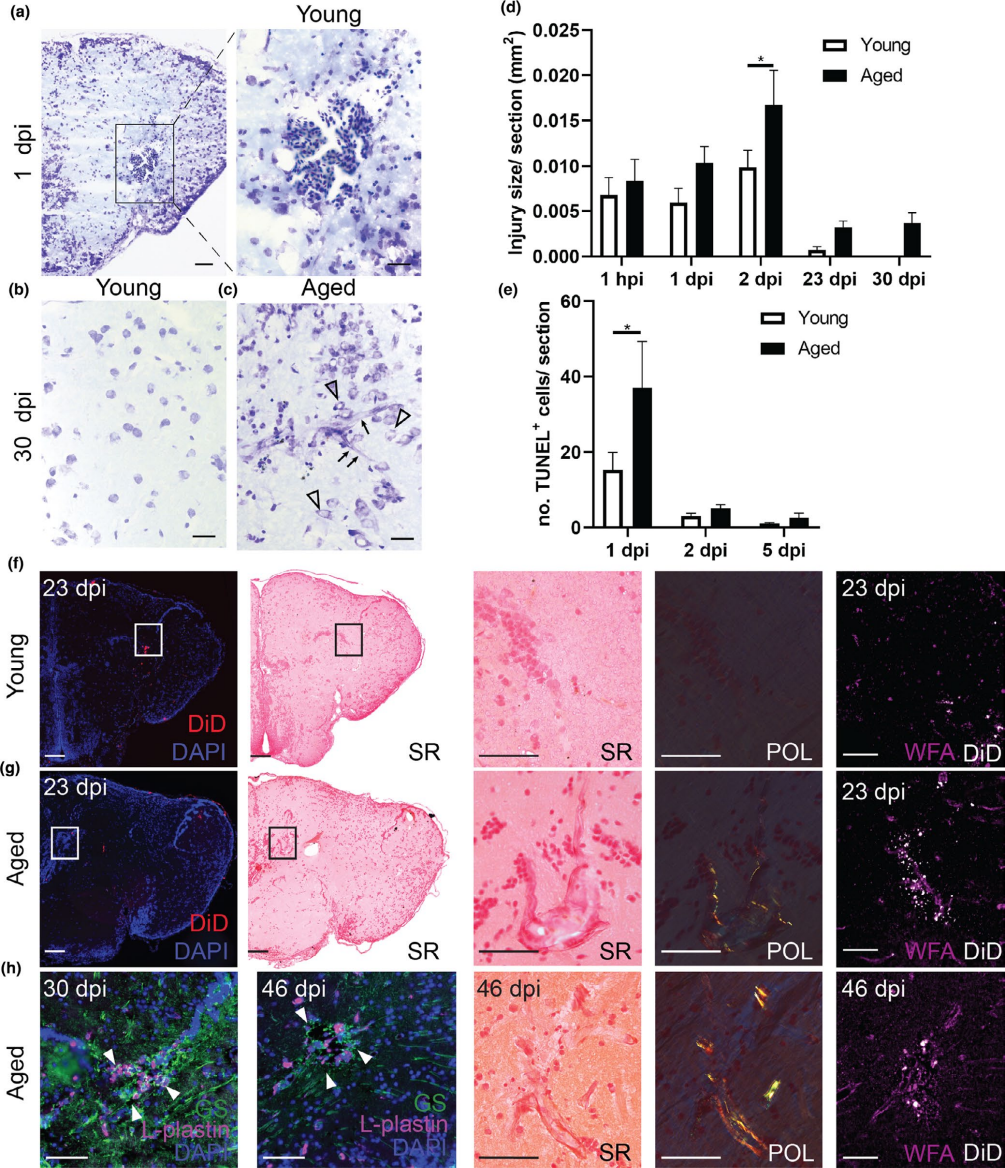
Aging impairs tissue recovery after brain injury by the induction of a glial scar. (a–c) Cresyl violet staining of coronal sections illustrate the injury site and tissue recovery after brain injury in the telencephalon of young (a,b) and aged killifish (c). At 30 dpi, the aged telencephalon still shows a tissue scar in the parenchyma (c), while the young telencephalon has no visible injury anymore (*n* ≥ 4). Cells (arrowheads) and blood vessels (arrows) near the tissue scar appear swollen (c). Scale bars: in a‐c: 50 µm; in insets: 20 µm. (d) Quantification of the injury surface area (in mm^2^) measured at 1 hpi and 1, 2, 23, 30 dpi, in young adult and aged fish. At 1 hpi, the size is similar, but toward 2 dpi the injury is significantly enlarged in aged fish. The injury is still visible at 23 and 30 dpi, time points at which young adult fish demonstrate extensive structural recovery. *p ≤ 0.05; two‐way ANOVA, followed by Sidak's multiple comparisons test. Values are mean ± SEM; *n* ≥ 4. (e) Countings of the absolute number of TUNEL^+^ apoptotic cells detected in the injured telencephalon at 1, 2, and 5 dpi reveal that apoptosis is significantly larger in aged fish at 1 dpi. *p ≤ 0.05; two‐way ANOVA, followed by Sidak's multiple comparisons test. Values are mean ± SEM; *n* ≥ 4. (f, g) Picro sirius red staining and WFA staining to, respectively, visualize collagen (using polarized (POL) light) and proteoglycan deposition at the injury site at 23 dpi in young (f) and aged (g) killifish (*n* ≥ 4). While young killifish do not have collagen fibers at the DiD‐positive injury site, aged killifish do. In addition, aged killifish have increased WFA staining at the scar tissue, indicating augmented deposition of proteoglycans. (h) Double staining for L‐plastin (red) and GS (green) with DAPI (blue) on coronal brain sections of young and aged killifish at 30 and 46 dpi (*n* = 4). At both time points, a cluster of L‐plastin^+^ microglia/macrophages fills the center of the glial scar, which is surrounded by GS^+^ RG fibers. Even at 46 dpi, collagen deposition and WFA staining is still visible and marks the glial scar in aged killifish. Scale bars in overview pictures: 100 µm; in insets: 50 µm. hpi, hours post‐injury; dpi, days post‐injury; SR, Picro sirius red; POL, polarized light; WFA, Wisteria Floribunda agglutinin; GS, glutamine synthetase; RG, radial glia. Panels in (a, b, and c) are also shown in Figure S2 to illustrate how the injury surface area was measured, and in Figure S1 to visualize the DiD dye at the site of injury

Standard histology revealed a packed blood clot that filled the injury site in the brain of young adult and aged killifish immediately upon injury (Figure 1a). In ensuing weeks, the parenchyma of the young adult telencephalon was able to structurally regenerate in a seamless fashion, showing a normal distribution of cells even at the DiD‐positive injury site, 23 to 30 days post‐injury (dpi) (Figures 1b and S1). In aged killifish on the contrary, the parenchyma showed a malformation, recognizable by swollen and irregularly‐shaped cells and blood vessels at 23 to 30 dpi (Figures 1c and S1G). We tested if the scar tissue, typically observed in aged killifish, was reminiscent of a mammalian glial scar. As the teleost telencephalon is devoid of parenchymal astrocytes (Grupp et al., 2010), we probed for the presence of microglia and the long bushy fiber of RGs (GS^+^) that span the entire parenchyma. At 23 and 30 dpi, a cluster of L‐plastin^+^ microglia/macrophages was present at the injury site, co‐localizing with the scar tissue and the DiD labeled cells in aged killifish (Figures 1h and S1). This cluster of microglia was surrounded by GS^+^ fibers of the RG, indicating that RGs were involved in glial scarring via the use of their bushy fiber (Figures 1h, S1 and S2). We performed Picro Sirius Red staining to visualize collagen fibers at the scar. While young killifish did not show any collagen deposition at their DiD‐positive injury site, yellow, green, and red collagen fibers were visible in aged killifish using polarized light (Figures 1f,g and S2). Via Wisteria floribunda agglutinin (WFA) staining, we also probed for the presence of glycoproteins within the extracellular matrix of the glial scar and aged fish indeed displayed a more pronounced WFA staining (Figures 1g and S2). Microglia, astrocytes (in our case GS^+^ RG fibers), collagen, and chondroitin proteoglycan deposition are typical hallmarks normally associated with mammalian glial scarring and—to our knowledge—never observed in the teleost injured telencephalon to this extent. To assess whether glial scarring was permanent, we analyzed fish at 46 dpi, at a time when half of the fish have died of old age. All remaining fish still showed glial scarring, as visualized by an L‐plastin^+^ microglia cluster, surrounded by RG fibers, collagen deposition, and intense WFA staining at 46 dpi (Figures 1h and S2). As such, these observations indicate that the glial scar is likely permanent in aged killifish.

In conclusion, stab‐wound injury could effectively be applied to compare the regeneration process between young and aged killifish brains. Aged killifish brains recovered incompletely and showed signs of permanent glial scarring, reminiscent of mammalian glial scarring.

### 2.3 A high inflammatory response exacerbates tissue recovery in aged killifish

**FIGURE 2:**
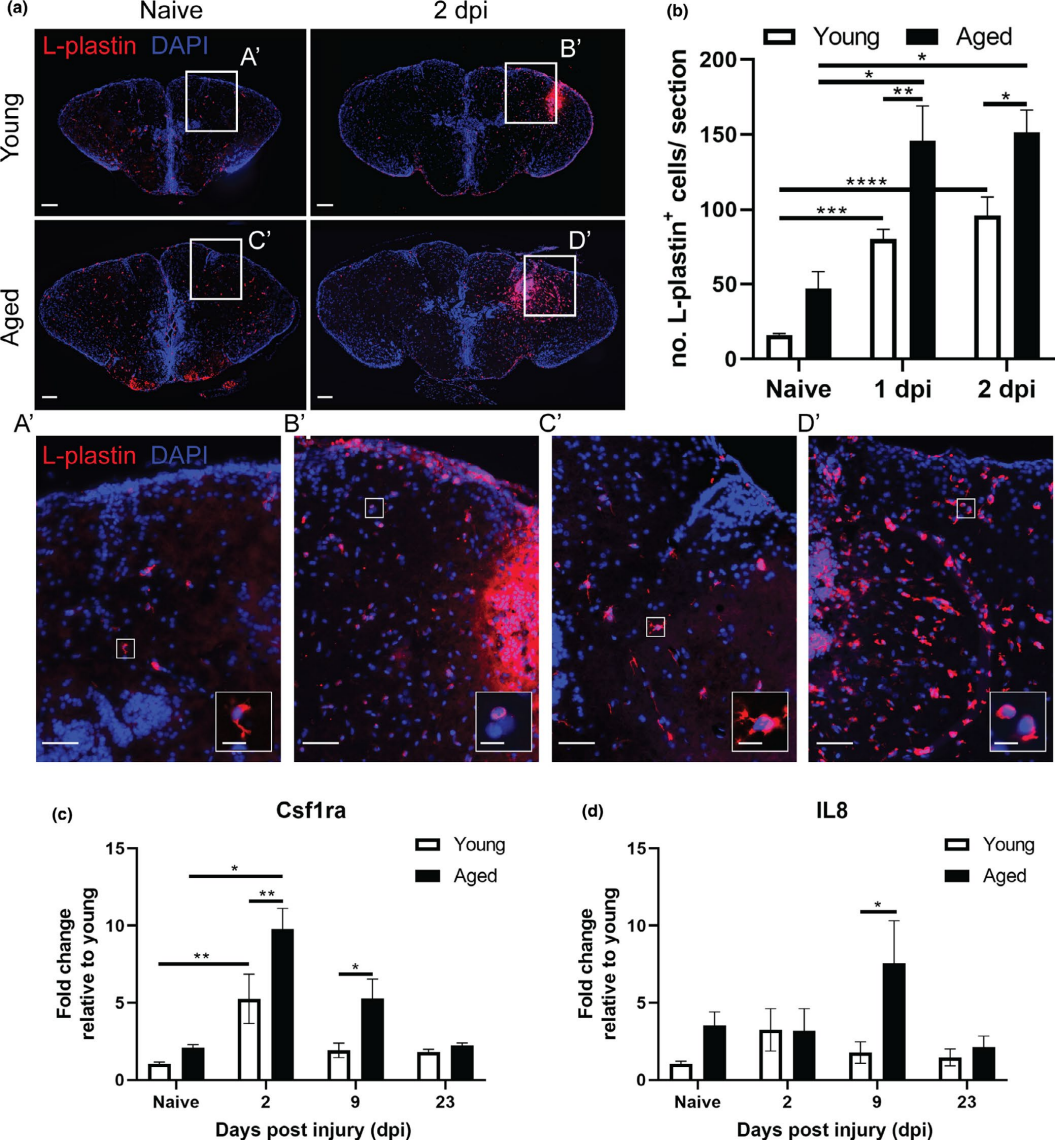
The injury‐induced inflammatory reaction is larger in the aged brain. (a) Staining for L‐plastin (red) with DAPI (blue) on coronal brain sections of young adult and aged, naive and 2 dpi killifish. The increase in L‐plastin^+^ inflammatory cells is more pronounced in aged injured fish (A’–D’). Higher magnification of individual L‐plastin^+^ microglia/macrophages are presented in the right bottom corner. Scale bars: in a and A’–D’: 100 µm; in boxed areas in A’–D’: 10 µm. (b) Absolute number of L‐plastin^+^ microglia/macrophages in young adult and aged telencephali in naive conditions and at 1 and 2 dpi. Both age groups show a significant increase in inflammatory cells early after brain injury, but the number of microglia/macrophages is significantly higher in aged fish at 1 and 2 dpi. (c, d) Relative expression values of inflammation markers *c* *sf1ra* (c) and *il* *8* (d) in young and aged telencephalic, in naive conditions and at 2, 9, and 23 dpi. Aged killifish show higher and prolonged expression of *c* *sf1ra* and *il* *8* after injury compared to young fish. *p ≤ 0.05, **p ≤ 0.01, ***p ≤ 0.001, and ****p ≤ 0.0001; one‐way ANOVA is used to compare naive fish to injured fish. Young: parametric one‐way ANOVA, followed by Dunnett's multiple comparisons test. Aged: non‐parametric Kruskal–Wallis test, followed by Dunn's multiple comparisons test. Two‐way ANOVA is used to compare young and aged fish, followed by Sidak's multiple comparisons test. Values are mean ± SEM; *n* ≥ 4. dpi: days post‐injury, Csf1ra: colony‐stimulating factor 1 receptor a, and IL8: Interleukin 8

To be certain that the stab‐wound injury created a comparable injury in young and aged animals, we studied the temporal dynamics of the surface size of the injury using (immuno)histology (Figure S3). One hour after injury, the size of the wound was comparable between young adult and aged killifish. By 2 dpi, the injury size was significantly enlarged in aged killifish, possibly due to differences in cell death, arrival of inflammatory cells and edema (Figure 1d). By counting the number of TUNEL^+^ apoptotic cells, we discovered that the aged injured brain contained more TUNEL^+^ apoptotic cells than young adult fish, already at 1 dpi (37±12.3 versus 15.3 ± 4.6; *p* = 0.0128, Figures 1e and S4). It thus seems that in an aged injured environment, cells were more vulnerable to secondary damage, which might be linked to an inflammatory state. Led by these results, we stained for the microglia/macrophage marker L‐plastin at 1 and 2 dpi. As expected, stab‐wound injury induced an acute inflammatory response reflected in an increased number of microglia/macrophages at 1 and 2 dpi in injured fish compared to naive fish (uninjured controls, Figure 2a,b). In addition, we detected more microglia/macrophages in aged injured killifish compared to young adult injured killifish, indicating a higher inflammatory response in aged killifish (Figure 2a,b). This may explain the higher number of apoptotic cells seen at 1 dpi, as well as why the size of the injury site increases toward 2 dpi in aged killifish. In addition, a fraction of these microglia/macrophages were PCNA^+^ (Figure S5), and thus proliferating. The higher inflammatory state of the aged injured brain was also reflected in the augmented expression of the macrophage marker colony‐stimulating factor 1 receptor a (*c* *sf1ra*) and the pro‐inflammatory marker interleukin 8 (*il* *8*). While *c* *sf1ra* and *il* *8* levels of young adult fish had dropped to baseline levels by 9 dpi, aged fish still showed high expression, suggesting the inflammatory reaction is prolonged in aged injured fish (Figure 2c,d).

### 2.4 Reactive proliferation is declined and delayed in aged injured killifish brains

For brain repair to be successful, new neurons should be generated from the available neuronal progenitor cell (NPC) pool. We counted the number of activated NPCs (SOX2^+^ PCNA^+^) and all NPCs (SOX2^+^) in the dorsal ventricular zone (VZ) (Figure S6) in function of age and time post‐injury.

**FIGURE 3:**
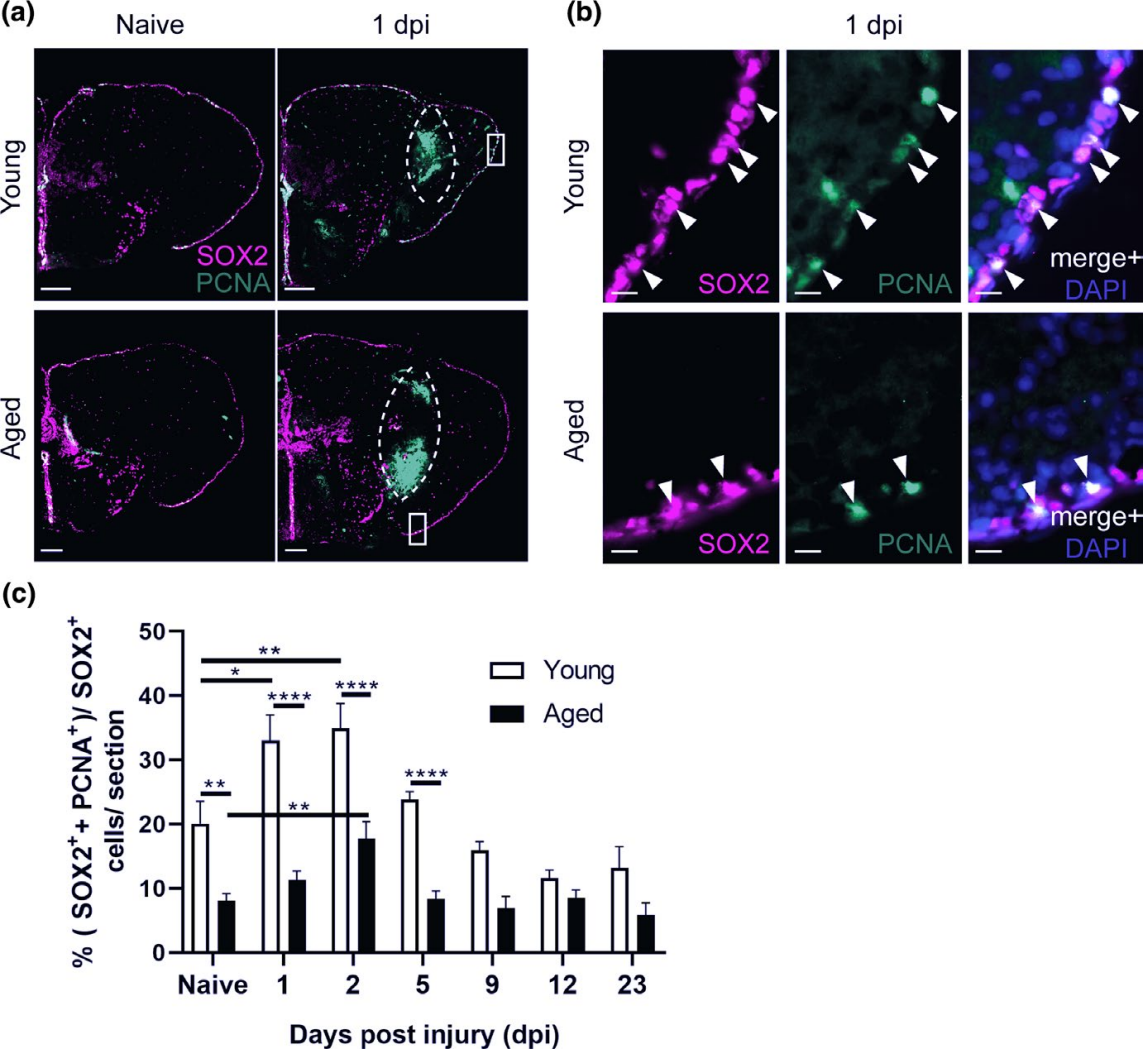
Aging diminishes neural progenitor cell proliferation in the ventricular zone of the killifish telencephalon, in naive conditions and in response to injury. (a, b) Double staining for SOX2 (magenta) and PCNA (green) with DAPI (blue) on coronal sections of young and aged killifish in naive conditions and at 1 dpi. The dashed lines encircle the site of injury filled with blood cells that autofluoresce in the green channel. (b) Magnifications of the boxed areas in a: Young adult fish have a higher percentage of proliferating progenitor cells (SOX2^+^ PCNA^+^ cells, arrowheads) compared to aged fish. Scale bars: in a: 100 µm;. in b: 10 µm. (c) Proportion of double‐positive SOX2^+^ PCNA^+^ dividing progenitor cells among SOX2^+^ cells (all progenitor cells) in young adult and aged telencephali in naive conditions and at 1, 2, 5, 9, 12, and 23 dpi. At all time points, young adult fish have a higher capacity for progenitor proliferation. For both ages progenitor proliferation peaks at 2 dpi. *p ≤ 0.05, **p ≤ 0.01, and ****p ≤ 0.0001; one‐way ANOVA is used to compare naive fish to injured fish, followed by Dunnett's multiple comparisons test. Two‐way ANOVA is used to compare young and aged fish at each time point, followed by Sidak's multiple comparisons test. Values are mean ± SEM; *n* ≥ 5, except for aged, 9 dpi: *n* = 4. dpi: days post‐injury

The percentage of dividing NPCs in the progenitor pool of aged naive fish was cleary lower than in young fish (8.1% ± 1 versus 20% ± 3.5; *p* = 0.0047, Figure 3), and this difference persisted upon injury. In young killifish, the injury‐induced proliferation of NPCs was significantly higher than in aged killifish (at 2 dpi: 34.9% ± 3.8 versus 17.8% ± 2.6, respectively; *p* < 0.0001, Figure 3). The increase occurred at 1 and 2 dpi, and declined back to normal levels from 5 dpi onward. In the aged killifish, the percentage was significantly higher at 2 dpi compared to naive aged fish, but it did not even reach the baseline levels of the young adult killifish. These results show that the capacity for NPC proliferation upon injury diminishes steeply with age, but that aged killifish still retain some capacity for NPC reactive proliferation. In addition, at 1dpi, we also found reactive proliferation of NPCs in the uninjured hemisphere of young adult killifish, but not in aged killifish. This observation might indicate that, in contrast to aged NPCs, young adult NPCs are still responsive to systemic effects (Figure S7).

### 2.5 Reactive proliferation is mainly supported by NGPs and not RGs

**FIGURE 4:**
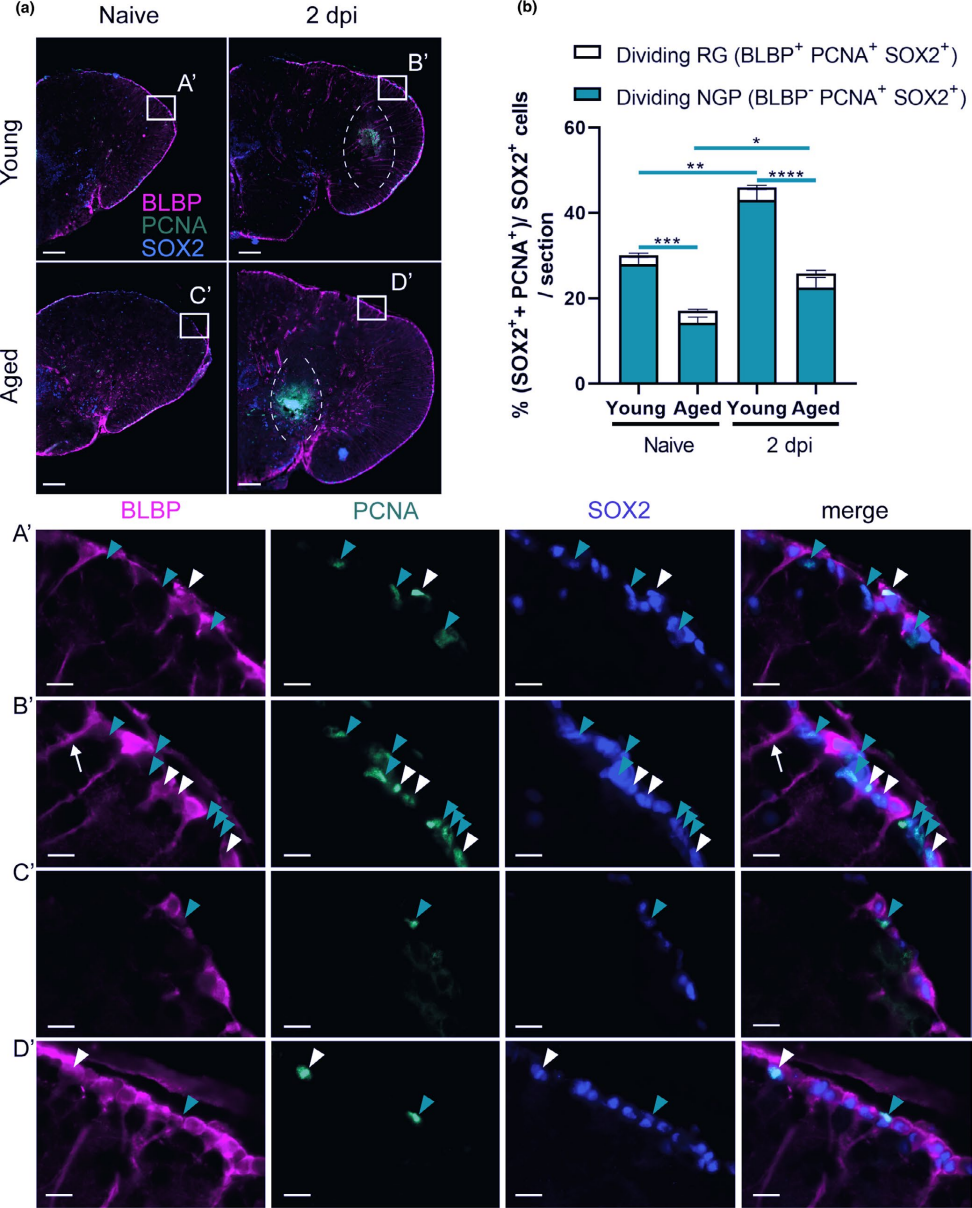
Aging reduces the proportion of dividing specialized NGPs, but not dividing common RGs. (a) Triple staining for BLBP (magenta), PCNA (green), and SOX2 (blue) on coronal sections of young adult and aged killifish in naive conditions and at 2 dpi. The dashed lines encircle the site of injury. Boxed areas are magnified in A’‐D’. White arrowheads depict triple‐positive BLBP^+^ SOX2^+^ PCNA^+^ dividing RGs, while turquoise arrowheads mark double‐positive BLBP^−^ SOX2^+^ PCNA^+^ dividing NGPs. (B’) Thicker RG fibers are noticed in injured fish (white arrow), indicative for glial activation. Young adult fish clearly have a higher percentage of dividing NGPs compared to aged fish. Dividing RGs are only observed in small amounts in the VZ. Scale bars: in a: 100 µm; in A’–D’: 10 µm. (b) Proportion of BLBP^+^ SOX2^+^ PCNA^+^ dividing RG and BLBP^−^ SOX2^+^ PCNA^+^ dividing NGPs over all SOX2^+^ cells (all progenitor cells) in the VZ of young and aged telencephali in naive and 2 dpi fish. Reactive progenitor proliferation is mainly supported by specialized NGPs and is highest in young adult fish. Aged fish have significantly lower percentages of dividing NGPs in both naive and injured conditions. *p ≤ 0.05, **p ≤ 0.01, ***p ≤ 0.001, and ****p ≤ 0.0001; Unpaired t test is used to compare naive fish to injured fish. Two‐way ANOVA is used to compare young and aged fish, followed by Sidak's multiple comparisons test. Values are mean ± SEM; *n* ≥ 5. RG, radial glia; NGP, non‐glial progenitor; VZ, ventricular zone; dpi, days post‐injury

We investigated which of the two progenitor types support reactive proliferation in our injury model at 2 dpi, the time point at which reactive proliferation peaks independent of age (Figure 3). By immunohistochemistry for SOX2, PCNA, and BLBP, we could delineate the dividing RGs (SOX2^+^ PCNA^+^ BLBP^+^) from the dividing NGPs (SOX2^+^ PCNA^+^ BLBP^−^). Strikingly, we rarely observed dividing RGs (approximately 3% of all NPCs, Figure 4), although RG fibers had a swollen morphology after injury (Figure 4B'). Instead, we clearly observed many dividing NGPs in the naive and injured dorsal VZ of young and aged killifish, showing that NGPs are the most prominent cell type supporting adult neurogenesis in killifish (Figure 4). The percentage of dividing NGPs among all NPCs was significantly lower in aged compared to young adult fish (Figure 4b), both in naive (14.26% ± 1.328 versus 28% ± 1.6; *p* = 0.0005) and injured animals (22.5% ± 2.3 versus 43% ± 2.5; *p* < 0.0001), demonstrating that adult neuro(re)genesis declined upon aging.

Unlike in zebrafish, we provide evidence that injury‐induced reactive proliferation of progenitors is mainly supported by the NGPs in the killifish pallium. Of all dividing NPCs present in the young injured adult VZ at 2 dpi, 93.5% ± 0.7 could be assigned to NGPs, while only 6.5% ± 0.7 were of RG type (Table S1). This is in sharp contrast to young adult injured zebrafish where 86.5% ± 3.1% (*n* = 4) of dividing cells represented RGs at 3 dpi (Kroehne et al., 2011). In the aged injured killifish 87.7% ± 1.8 of all dividing NPCs were NGPs, while 12.3% ± 1.8 were RG (Table S1). Aged injured killifish thus showed a higher percentage of dividing RG among all dividing cells compared to young adult injured killifish. A similar observation was made for naive conditions (Table S1). It appears that aged killifish try to compensate their reduced capacities by activating the quiescent RG pool.

Taken together, killifish NGPs represent the most potent progenitor type driving the proliferative response to injury, but their number declines significantly with age when RGs appear more activated, albeit still low in respect to NGPs.

### 2.6 High senescent cell burden and high expression of cell cycle inhibitors typify the reduced reactive proliferation of progenitor cells in aged killifish

**FIGURE 5:**
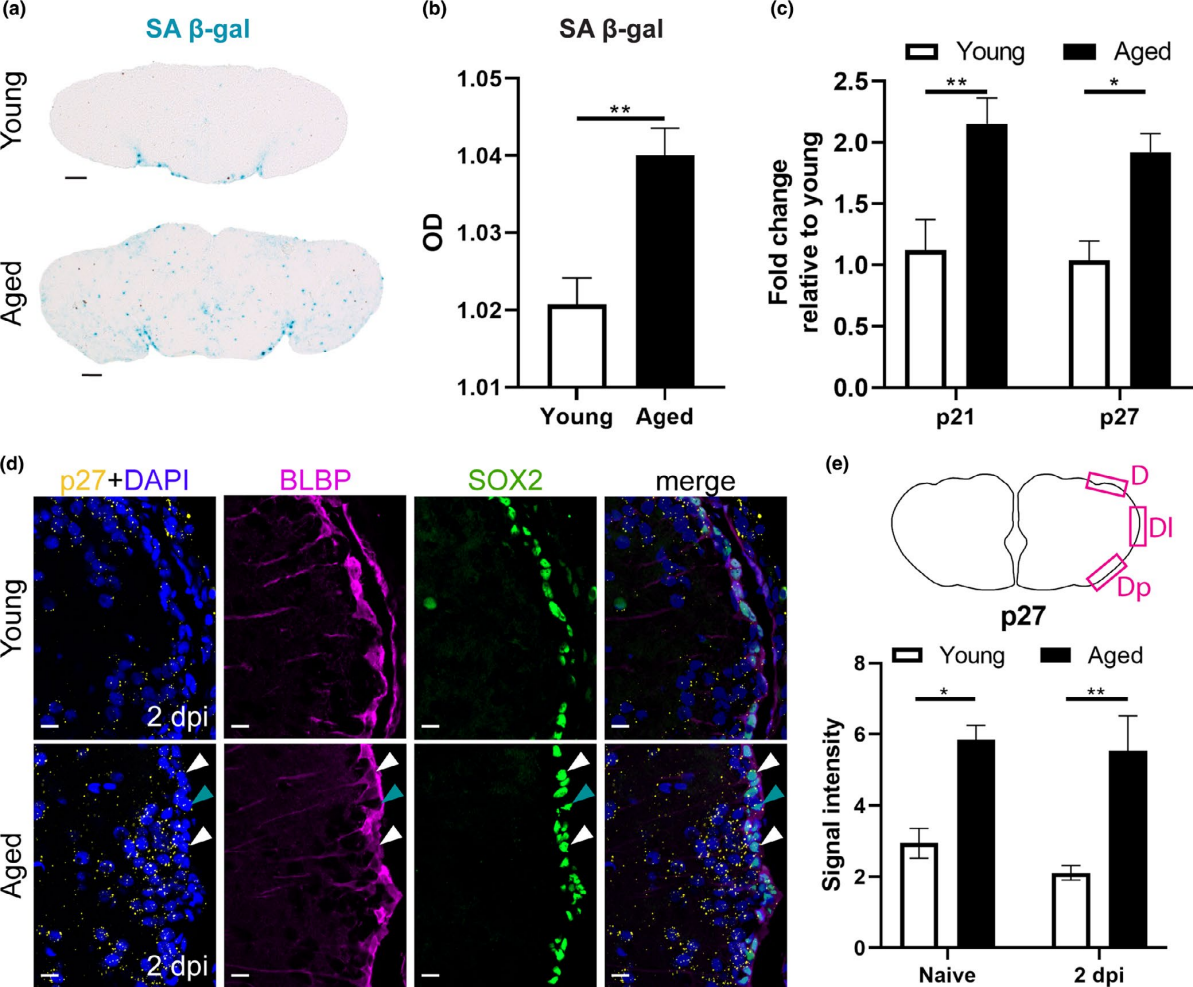
A high senescent cell burden and high expression of cell cycle inhibitors typify the reduced reactive proliferation of progenitor cells in aged killifish. (a) SA β‐gal staining on coronal sections of young adult and aged naive telencephali. (b) Optical density (OD) measurement of SA β‐gal staining of young and aged naive killifish reveals a higher staining intensity in aged killifish, indicating a higher senescent cell burden. Unpaired t test. Values are mean ± SEM; *n* ≥ 4. (c) Relative expression values of the cell cycle inhibitors *p21* and *p27* are higher in aged telencephali compared to young telencephali. Two‐way ANOVA is used to compare young and aged fish, followed by Sidak's multiple comparisons test. Values are mean ± SEM; *n* ≥ 4. (d) Fluorescent *in situ* labeling of *p27* (yellow, HCR), combined with double staining for BLBP (magenta) and SOX2 (green) with DAPI (blue) on coronal sections of young and aged telencephali in naive conditions and at 2 dpi. The expression of cell cycle inhibitor *p27* is higher in aged killifish and seems to be localized to RGs (white arrowheads), NGPs (turquoise arrowheads), and newborn neurons (cells lying close to the VZ). (e) *p27* signal intensity was measured in a set region of interest in the dorsal (D), dorso‐lateral (Dl), and dorso‐posterior (Dp) portion of the VZ. Aged killifish show a higher signal intensity of *p27* compared to young individuals, suggesting increased expression of *p27*. *p ≤ 0.05, **p ≤ 0.01; unpaired t test is used to compare naive to 2 dpi fish. Two‐way ANOVA is used to compare young and aged fish, followed by Sidak's multiple comparisons test. Values are mean ± SEM; *n* = 3. SA β‐gal, senescence‐associated β galactosidase; OD, optical density; RG, radial glia; NGP, non‐glial progenitor; VZ, ventricular zone; dpi, days post‐injury; D, dorsal; Dl, dorso‐lateral; Dp, dorso‐posterior

Since we discovered that the aged regeneration capacity was characterized by a very low ability for reactive proliferation, we investigated if this was linked to a higher incidence of senescence or a higher expression of cell cycle inhibitors in aged killifish. The senescence‐associated β‐galactosidase (SA β‐gal) assay visualizes increased lysosomal β‐galactosidase activity, which is typically associated with senescent cells (Lee et al., 2006). Using this assay, we revealed increased SA β‐gal staining in aged fish (*p* = 0.0064, Figure 5a,b). The senescent state of the aged killifish was also confirmed by the elevated expression of the two cell cycle inhibitors *p21* and *p27* (p21: *p* = 0.0044, p27: *p* = 0.0132, Figure 5c) measured with RT‐qPCR. In order to validate that the expression of the cell cycle inhibitors was localized to RGs or NGPs, we used hybridization chain reaction (HCR v3.0), a technique based on the use of DNA probe pools carrying DNA initiators. When fluorophore‐labeled DNA hairpins are added, the DNA initiators trigger chain reactions so that the hairpins will form tethered polymers and boost the fluorescent signal (Choi et al., 2016, 2018). Via HCR, we fluorescently labeled *p27* transcripts on coronal sections of the young and aged telencephalon in naive conditions and at 2 dpi. By double labeling with SOX2 and BLBP, we could distinguish between *p27*‐positive RG (SOX2^+^ BLBP^+^) or NGPs (SOX2^+^ BLBP^−^). We discovered that aged fish had elevated *p27* expression levels, even at 2 dpi, compared to young fish (naive: *p* = 0.0147, 2 dpi: *p* = 0.0059, Figure 5e). Labeling of *p27* transcripts was present in both RGs and NGPs, and in cells lying close to the VZ (Figure 5d).

All together, these results point toward increased senescence and expression of cell cycle inhibitors in RGs and NGPs as potential underlying causes of the reduced reactive proliferation capacity of aged killifish, even after injury.

### 2.7 A declined number of newborn neurons reaches the injury site in aged killifish

**FIGURE 6:**
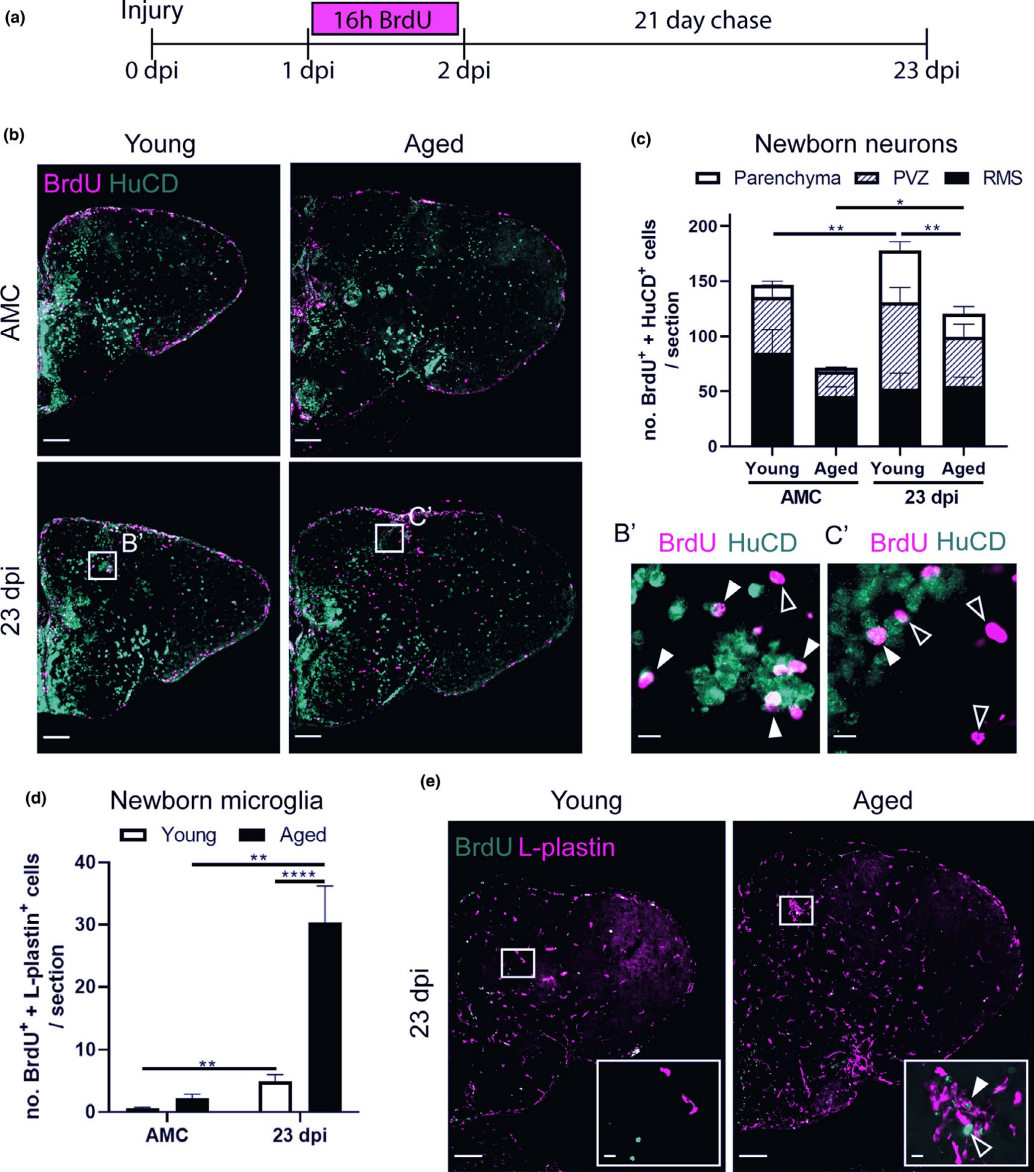
Aged killifish generate less newborn neurons but more newborn microglia/macrophages in the parenchyma of the telencephalon compared to young adult killifish. (a) Experimental set up: Day 0: time of stab‐wound injury; from 1 to 2 dpi: injured fish and AMCs are placed in BrdU water for 16 h: BrdU will be incorporated in the DNA of dividing cells and passed on to the progeny upon each cell division. 23 dpi: regeneration is completed in young fish, all brain samples of young and aged, AMC and injury conditions, are collected for cell analysis (illustrated in b‐e). (b) Double staining for BrdU (magenta) and HuCD (green). Boxed areas are magnified in B’,C’. White closed arrowheads depict double‐positive BrdU^+^ HuCD^+^ newborn neurons; open arrowheads indicate newborn single‐positive BrdU^+^ cells of unknown cell type. While the BrdU signal overlaps with HuCD (neuronal marker) in young adult fish, representing newborn neurons, aged fish mostly have BrdU^+^ cells lying next to HuCD^+^ neurons, suggesting these cells are another cell type. Scale bars: in b: 100 µm; in B’,C’: 10 µm. (c) Number of BrdU^+^ HuCD^+^ newborn neurons in the parenchyma, PVZ of the dorsal pallium and near the RMS. In the parenchyma, significantly lower numbers of newborn neurons are present in aged injured compared to young adult injured fish. (d) Number of BrdU^+^ L‐plastin^+^ newborn microglia/macrophages in the parenchyma. Aged injured fish generate large numbers of newborn microglia/macrophages by 23 dpi. *p ≤ 0.05, **p ≤ 0.01, and ****p ≤ 0.0001; unpaired t test or non‐parametric Mann–Whitney test is used to compare AMC to 23 dpi fish. Two‐way ANOVA is used to compare young and aged fish, followed by Sidak's multiple comparisons test. Values are mean ± SEM; *n* ≥ 5. (e) Double staining for BrdU (green) and L‐plastin (magenta). Boxed areas are magnified in the right corner of each panel. A cluster of L‐plastin^+^ microglia/macrophages is visible in the parenchyma of aged injured but not young injured fish, at 23 dpi. White closed arrowhead depicts a BrdU^+^ L‐plastin^+^ newborn microglia/macrophage, inside this cluster. Open arrowhead points to a green autofluorescent blood cell recognizable by its oval shape and visible nucleus. Scale bars: in e: 100 µm; in boxed areas: 10 µm. AMC, age‐matched control; RMS, rostral migratory stream; PVZ, periventricular zone; dpi, days post‐injury

To elucidate whether reactive NGPs lead to the production of newborn neurons that can migrate to the injury site for replacement of the lost cell types, we designed a BrdU pulse chase experiment. In between 1 and 2 dpi, when reactive proliferation is most intense (Figure 3), young and aged injured and age‐matched control (AMC) killifish were subjected to 16 h of BrdU water, labeling all cells passing through S‐phase (Figure 6a). After a 21‐day chase period, a time window matching maximal recovery in young adult fish, we visualized the progeny of these cells via immunostaining for BrdU and HuCD. HuCD is a pan‐neuronal marker, expressed in both immature and mature neurons. As such, BrdU^+^ HuCD^+^ neurons, that represent the progeny of the dividing NGPs, were visualized at 23 dpi (Figure 6b).

Our data reveal impaired production and migration of newborn neurons in aged killifish at 23 dpi. In naive fish, we could hardly detect newborn neurons that had migrated into the parenchyma (10.9 ± 3.3 for young adult and 3.6 ± 0.6 for aged fish) (Figure 6c). Many newborn neurons in the periventricular zone (PVZ) of the dorsal pallium and near the rostral migratory stream (RMS) (Figure S6) migrate only 1–2 cell diameters away from the ventricular stem cell zones, which is typical for constitutive neurogenesis in adult teleosts (Adolf et al., 2006). Injured killifish on the contrary generated more newborn neurons that migrated into the injured parenchyma. This number was significantly higher in young adult killifish than in aged killifish (47 ± 8 versus 20.8 ± 6.5; *p* = 0.005) (Figure 6c).

Most likely the impaired replenishment of newborn neurons in aged injured animals is caused by a declined production of neurons, yet also the failure of these neurons to migrate toward the injury site through the aged non‐permissive environment. Indeed, we confirmed that aged injured killifish produce large numbers of newborn microglia/macrophages (BrdU^+^ L‐plastin^+^) by 23 dpi (aged killifish: 30.3 ± 5.9 versus young adult killifish: 4.9 ± 1.1; *p* < 0.0001), what most likely contributes to an inflammatory non‐permissive environment (Figure 6d,e).

### 2.8 Aging hampers the replenishment of progenitors in the VZ after injury

**FIGURE 7:**
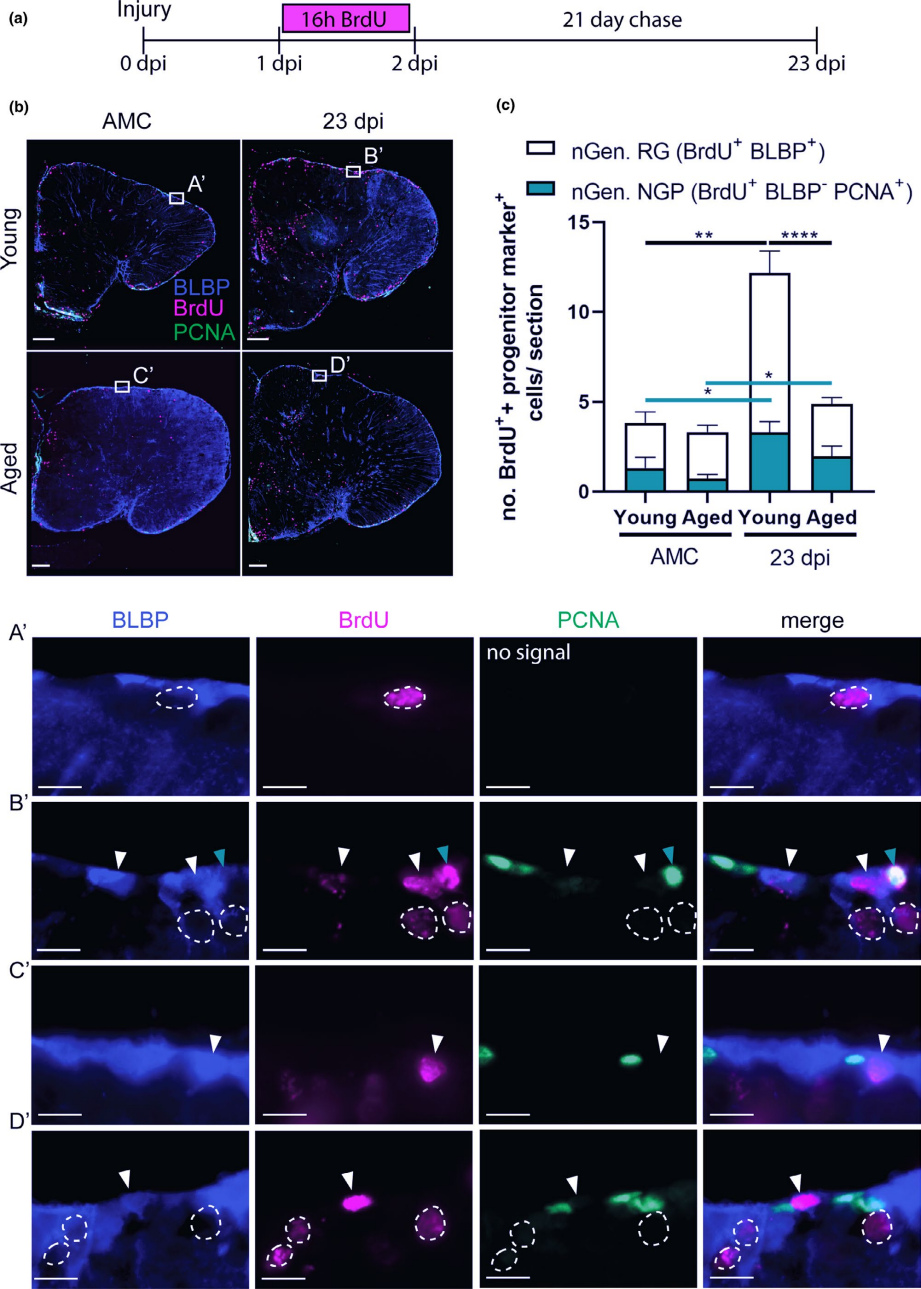
Aging hampers the replenishing of progenitors in the VZ after injury. (a) Experimental set up: Day 0: time of stab‐wound injury; from 1 to 2 dpi: injured fish and AMCs are placed in BrdU water for 16 h: BrdU will be incorporated in the DNA of dividing cells and passed on to the progeny upon each cell division. 23 dpi: regeneration is completed in young fish, all brain samples of young and aged, AMC and injury conditions are collected for cell analysis (as illustrated in b). (b) Triple staining for BLBP (blue), BrdU (magenta), and PCNA (green). Boxed areas are magnified in (A’‐D’). White arrowheads depict double‐positive BLBP^+^ BrdU^+^ newly generated RGs. Turquoise arrowheads mark double‐positive BLBP^−^ BrdU^+^ PCNA^+^ newly generated NGPs. Newly generated RGs, and to a lesser extent newly generated NGPs, are produced in the VZ upon injury. Scale bars: in b: 100 µm; in A’‐D’: 10 µm. (c) Number of BLBP^+^ BrdU^+^ newly generated RGs and BLBP^−^ BrdU^+^ PCNA^+^ newly generated NGPs in the VZ of young and aged telencephali in AMC and at 23 dpi. Significantly more newly generated progenitors are produced after injury. Young adult fish create significantly more newly generated RGs compared to aged killifish at 23 dpi. This difference is not significant for newly generated NGPs. *p ≤ 0.05, **p ≤ 0.01, and ****p ≤ 0.0001; unpaired t test or non‐parametric Mann–Whitney test is used to compare AMC to 23 dpi fish. Two‐way ANOVA is used to compare young and aged fish, followed by Sidak's multiple comparisons test. Values are mean ± SEM; *n* ≥ 5. RG, radial glia; NGP, non‐glial progenitor; AMC, age‐matched control; VZ, ventricular zone; dpi, days post‐injury; nGen, newly generated

Stab‐wound injury disrupts the parenchyma of the dorsal telencephalon, but also the VZ. Hence, the dorsal VZ needs to be replenished with newly generated NGPs and RGs. We applied BrdU pulse chase labeling to investigate whether dividing progenitors, labeled between 1 and 2 dpi, give rise to new progenitors by 23 dpi, the time point where regeneration is complete in young adult fish (Figure 7a). We zoomed in on the two major progenitor classes: (1) newly generated RGs (BrdU^+^ and BLBP^+^) and (2) newly generated dividing NGPs (BrdU^+^, PCNA^+^, and BLBP^−^). In the highly proliferative NGPs, the BrdU signal will be diluted with each cell division and eventually gets lost. This assay thus rather describes the progeny of low proliferative progenitors that is the RGs in killifish. Indeed, in all conditions, we found a larger number of newly generated RGs than NGPs. RGs, labeled between 1 and 2 dpi, thus seem to generate new RGs via gliogenic divisions. Whether RGs also gave rise to NGPs or vice versa remains elusive and an interesting research question for future studies. Of note, we also detected a low number of BrdU^+^ cells in the VZ that were PCNA^−^ and BLBP^−^, which we thus could not identify as RG or NGP. We, however, realize that these cells could possibly represent a quiescent progenitor type or newborn neuroblasts, lying closely to the VZ.

Independent of the progenitor class, we observed that naive fish showed negligible amounts of newborn RG and NGPs. In injured brains, clearly more newly generated progenitors are present in the dorsal VZ, albeit much less in aged compared to young adult fish (newly generated RG: 2.9 ± 0.4 versus 8.9 ± 1.2; *p* < 0.0001; newly generated NGP: 2 ± 0.6 versus 3.3 ± 0.6; *p* = n.s., Figure 7b,c).

Remarkable, the dividing progenitor pool was replenished to baseline levels during the 21‐day chase for each specific age (Figure S8). For both progenitor classes, a similar number of dividing cells were observed compared to their respective AMCs (Figure S8). Aged fish, however, still had a lower number of dividing NGPs at 23 dpi compared to young adult animals. The number of dividing RGs was very low for both ages.

Taken together, these results indicate that aged killifish are less efficient in replenishing the dorsal VZ after stab‐wound injury compared to young adults. Furthermore, generation of neurons and progenitors does not exhaust the available dividing progenitor pools during a short 21‐day chase period, suggesting a great capacity of killifish for progenitor cell self‐renewal and neuro(re)genesis, especially in the young adult killifish.

## 3 DISCUSSION

We designed and benchmarked an easy‐to‐use and adequate TBI model to study neuroregeneration and uncover the impact of aging on brain repair in the fastest‐aging teleost laboratory species, *N*. *furzeri*. We discovered an age‐related decline in proliferation and injury‐related proliferative response of neurogenic progenitors. The NGP population is most active and responds most prominently to injury. Glial scarring and a strong, prolonged inflammatory reaction also typify the aged condition post‐injury, while young adult brains regenerate in a seamless manner. Taken together, our results provide a validated animal model for future studies to unveil underlying mechanisms driving the loss of full neuroregenerative capacity upon aging.

Stab‐wound injuries are reliable, effective injury models and require no specialized equipment, which makes them applicable in a broad range of laboratories. They allow studying a vast array of pathological conditions, since they elicit a clear multicellular read‐out, for example, disruption of the blood–brain barrier (BBB), inflammation, and astrogliosis. Stab‐injuries are often used in mice as TBI model (Frik et al., 2018; Hashimoto et al., 2018). They have also been extensively characterized in the zebrafish telencephalon (Ghaddar et al., 2021). The benefit of translating this model to the killifish is the implementation of the factor age to investigate its influence on the high neuroregenerative capacity, typically associated with teleost species. Surgery can be performed under 5 minutes, no specialized equipment is required and since teleost fish are regeneration‐competent vertebrates, full recovery is established within 23–30 days in young adult killifish (data presented here) and adult zebrafish (Ayari et al., 2010; Kishimoto et al., 2012; Kroehne et al., 2011).

The current study provides evidence that the killifish telencephalon is subjected to age‐associated stem cell exhaustion, one of the 9 hallmarks of aging (López‐Otín et al., 2013). Far less proliferating progenitors, newborn neurons and newly generated progenitors were counted in aged killifish, even after injury, which is in line with other reports in killifish (Tozzini et al., 2012) and zebrafish (Bhattarai et al., 2017; Edelmann et al., 2013). The reduced proliferation of NPCs in aged killifish was linked to increased cellular senescence and elevated expression of cell cycle inhibitors *p21* and *p27*. The expression of *p27* was localized to RGs, NGPs and to cells lying in the PVZ of the adult telencephalon, presumably neuroblasts. The high expression of *p27* in aged killifish RGs and NGPs thus hinders reactive proliferation after injury and ultimately overall repair. In addition, the RGs and NGPs were likely also extrinsically influenced by the senescence‐associated secretory phenotype (SASP) of senescent cells observed in the aged telencephalon. The SASP includes the secretion of chemokines, cytokines, and proteases among others, which is thought to create an unfavorable environment for tissue repair (Coppé et al., 2010; de Keizer, 2017; Krause et al., 2017).

One striking difference to other teleosts is that the reactive proliferation in killifish is related to the specialized NGPs, instead of the typical RGs that support zebrafish neuroregeneration (Baumgart et al., 2012; Kroehne et al., 2011). RG division is very low in killifish, even after injury. It was recently discovered that RGs enter a Notch3‐dependent quiescence already in larval stages. Whenever these RGs are reactivated, their division is mainly of gliogenic nature (Coolen et al., 2020). The low division of RGs in the killifish brain is reminiscent of adult neural stem cells (NSCs) in the SGZ and SVZ of mice. Here, GFAP^+^ NSCs are mostly quiescent and use predominately asymmetric divisions to create a new NSC and a transient amplifying progenitor. They can, however, also use symmetric divisions for self‐renewal (Daynac & Petritsch, 2017). Indeed, we and others discovered that killifish RGs are mostly quiescent, have low division potential, and can also self‐renew (Coolen et al., 2020). Whether killifish RGs can also generate NGPs via asymmetric division the way mammalian NSCs create transit‐amplifying cells remains, however, elusive. There are some similarities between NGPs and mammalian transient amplifying progenitors. Both progenitor types have a higher division rate compared to NSCs/RGs and are responsible for the production of newborn neurons (neuroblasts) (Coolen et al., 2020; Daynac & Petritsch, 2017). In the SVZ, generation of neurons happens via symmetric divisions, directly creating two neuroblasts from one transit‐amplifying progenitor (Daynac & Petritsch, 2017). NGPs on the contrary also appear to have a lot of self‐renewing capacity (Coolen et al., 2020). As such, NGPs might represent a separate progenitor lineage, created early in brain development, that is self‐sustainable while the RGs become quiescent upon adulthood. In the present study, we also discovered that the percentage of dividing RGs among all dividing cells was doubled in aged killifish, although still low in respect to the percentage of dividing NGPs. We hypothesize that when NGPs become exhausted with increasing age, more RGs become activated as a replenishing strategy. This would imply that RGs give rise to NGPs, which remains unexplored to date. Taken together, our results urge for dedicated lineage tracing from these two progenitor pools. Insights into the self‐renewal capacities of both progenitor types might unravel key concepts of stem cell biology and division mode necessary for successful neuroregeneration.

Notwithstanding the low percentage of dividing progenitors, aged killifish are still able to produce a neurogenic response upon injury. They generate a considerable number of newborn neurons, albeit much less than young adult killifish. Few neurons reach the injury site in the parenchyma, suggesting migration is also impacted by aging. It remains to be investigated if these neurons are still capable of maturing and integrating in the existing aged circuit, and if some degree of improved functional outcome can be established. The massive surge in microglia/macrophages at the injury site in aged killifish creates chronic inflammation as also confirmed by the high and prolonged expression of *c* *sfr1a* en *il* *8* in our study. Such sustained inflammation renders the parenchyma unsuitable for neuron migration, maturation, and integration, or may even drive the newborn neurons into apoptosis. This is similar to mammals, in which a large portion of the injury‐induced neurons will die due to the pathological environment (Grade & Götz, 2017; Turnley et al., 2014). We predict the chronic inflammation to be partly caused by influx of blood‐derived macrophages. Aging renders the BBB weak, enabling more macrophages to pass the BBB in aged brains (Montagne et al., 2015; Verheggen et al., 2020). This will increase the inflammatory response and exacerbate damage. Also in the aged zebrafish telencephalon, increased numbers of ramified, but not round, microglia were discovered after amyloidosis (Bhattarai et al., 2017). The elevated and prolonged expression of *c* *sf1ra* is of particular interest since Csf1ra is inflicted in macrophage‐mediated scar deposition in the zebrafish heart (Bevan et al., 2020). Indeed, aged killifish showed permanent glial scarring at the injury site, which is—to our knowledge—never witnessed before in other brain regeneration studies working with teleosts (Ghaddar et al., 2021). The glial scar, only seen in the aged killifish telencephalon, was characterized by a cluster of microglia surrounded by RG fibers and proteoglycan and collagen deposition. In mammals, the glial scar acts as a physical barrier consisting of reactive astrocytes, NG2 glia, and microglia. The glial scar preserves tissue integrity by repairing the BBB and blocking the influx of fibrotic cells and blood‐derived macrophages, yet also represents a physical barrier for axonal outgrowth, thereby restricting neural circuit integration and overall brain repair (Adams & Gallo, 2018). We hypothesize that also in aged injured killifish, the glial scar is preventing newborn neurons to integrate, thereby impeding successful brain recovery. The local presence of RG fibers, expressing high amounts of GS, suggests a function of RGs in glial scar formation. GS is known to convert glutamate into glutamine, reducing toxic extracellular glutamate levels (Zou et al., 2010). Its presence at the glial scar could indicate that high glutamate toxicity levels were still present in aged killifish. The cells surrounding the scar tissue indeed had a swollen morphology, typical for cytotoxicity (Liang et al., 2007).

Since our data predict that RGs do not support the production of new neurons in the killifish brain, the question remains what role RGs play after injury. At 2 dpi, RG fibers appeared swollen in injured killifish, suggesting that they do respond to the insult. Considering that many of the mammalian astrocyte‐specific genes (GLT‐1, BLBP, GS, GLAST, aldh1l1, GFAP, Vimentin, and S100β) are also expressed by teleost RGs (Chen et al., 2020; Coolen et al., 2020; März et al., 2010), a similar function is highly likely. In mammals, RGs act as neural stem cells early in development and support neurogenesis. Later on, most RGs leave the ventricular zone—the mammalian stem cell zone—and become “star‐shaped astrocytes” (Kriegstein & Alvarez‐Buylla, 2009). “Star‐shaped astroglia” have previously been described in the zebrafish spinal cord (Kawai et al., 2001), but are yet to be found in the teleost telencephalon. New evidence was recently provided that teleost “astroglia” resemble mammalian astrocytes more than once thought. In the larval zebrafish spinal cord, they are in close association with synapses, exhibit tiling and calcium signaling dynamics, and have a long bushy morphology that expands during development (Chen et al., 2020). In the zebrafish larval brain, interplay between astroglia and neurons during epileptic seizures was discovered, as well as large gap junction‐coupled glial networks (Diaz Verdugo et al., 2019). They can communicate with neurons and induce passivity in futile behavior paradigms (Mu et al., 2019). It thus seems that teleost RGs keep their developmental morphology into adulthood, but adopt several functions typically associated with mammalian astrocytes (Wahis et al., 2021). This might also explain why teleosts have such impressive regenerative abilities as their astroglia resemble more the “developmental” mammalian RGs, which makes them highly neurogenic into adulthood. The killifish thus represents a promising vertebrate model to unravel the function of the teleost “astroglia” because the delineation between RG stem cell properties and astrocyte properties seems more distinguished than in any other teleost model. How these killifish “astroglia” respond to injury and behave upon brain aging are intriguing research questions for the future.

Altogether, we expose the aged killifish to model low regenerative abilities similar to adult mammals. The aged neuroregenerative response is characterized by glial scarring, secondary damage, aggravating inflammation, reduced proliferation of stem cells, and reduced production of new neurons. Our model will therefore be highly useful to elucidate how to reverse the aging state of the brain in order to reinstate a high neuroreparative strategy as occurring in young adult killifish. Such insights will hopefully play a pivotal role in boosting neurorepair in mammals in the near future.

## 4 EXPERIMENTAL PROCEDURES

### 4.1 Fish strain and housing

All experiments involved adult (6‐week‐ and 18‐week‐old) female African turquoise killifish (*Nothobranchius furzeri*), inbred strain GRZ‐AD (kind gift by Prof. Dr. L. Brendonck and Dr. T. Pinceel; originating from the Biology of Ageing, Leibniz Institute for Age Research, Jena, Germany). Breeding pairs were housed in 8 L aquaria and experimental fish kept in 3.5 L aquaria in a ZebTEC Multi‐Linking Housing System (Tecniplast). One male was housed with 3 females under standardized conditions; temperature 28°C, pH 7, conductivity 600 μs, 12 h/12 h light/dark cycle, fed twice a day with *Artemia salina* and *Chironomidae* mosquito larvae (Ocean Nutrition). Breeding pairs were given sandboxes for spawning. Fertilized eggs were collected once a week and washed with methylene blue solution (03978, Sigma‐Aldrich, 0.0001% in autoclaved system water) for 5 min. Eggs were bleached 2× 5 min in 1% hydrogen peroxide, diluted in autoclaved system water. Eggs were again washed 4× 5 min with methylene blue solution. Eggs were stored on moist Jiffy‐7C coco substrate plates (Jiffy Products International AS, Norway) at 28°C with a 12 h/12 h light/dark cycle for three weeks in a custom‐made incubator. Embryos that reached the “Golden Eye stage” (Polačik et al., 2016) were hatched in a small volume of ice‐cold Humic acid solution (53680, Sigma‐Aldrich, 1 g/L in system water) with continuous oxygenation. Larvae were raised at 26°C, and half of the water was changed daily for one week. Hereafter, larvae were transferred to 3.5 L aquaria and fed daily with *Artemia salina* until 3 weeks post hatching. For lifespan experiments, 4 *N*. *furzeri* females (strain GRZ‐AD) were group‐housed. In total, 48 fish were daily monitored for survival during 261 days (maximal recorded lifespan). Based on these results, two age groups were determined, 6‐week‐old (100% survival, young adult) and 18‐week‐old killifish (76.6% survival, aged, Figure S1A). All experiments were approved by the KU Leuven ethical committee in accordance with the European Communities Council Directive of September 22, 2010 (2010/63/EU) and the Belgian legislation (KB of May 29, 2013).

### 4.2 Stab‐wound injury

Fish were anesthetized in 0.03% buffered tricaine (MS‐222, Sigma‐Aldrich), diluted in system water, and placed in a cold moist sponge. Scales, skin, and fat tissue were removed to visualize the skull. A custom Hamilton 33‐Gauge needle was pushed through the skull into the dorsal pallium of the right telencephalon, causing a brain lesion of approximately a depth of 500 μm (Figure S1, Video S1). The eyes flanking the telencephalon were used as landmarks. The needle was dipped in Vybrant DiD cell‐labeling solution (V22887, Thermo Fisher Scientific) to easily reconstruct entrance and needle track on sections (Figure S1, Video S1). Fish were placed in fresh system water to recover (Video S1).

### 4.3 BrdU labeling

To label dividing cells and their progeny, fish were placed in 5‐Bromo‐2’‐deoxyuridine (BrdU, B5002‐5G, Sigma‐Aldrich) water (7.5 mM in system water) for 16 h between 1 and 2 days post‐injury. After the pulse, fish were placed in fresh system water for a chase period of 21 days.

### 4.4 Tissue fixation and processing

Fish were euthanized in 0.1% buffered tricaine and perfused via the heart with PBS and 4% paraformaldehyde (PFA, 8.18715, Sigma‐Aldrich, in PBS). Brains were extracted and fixed for 12 h in 4% PFA at 4°C, washed 3× in PBS, and embedded in 30% sucrose, 1.25% agarose in PBS. Ten µm‐thick coronal sections were made on a CM3050s cryostat (Leica), collected on SuperFrost Plus Adhesion slides (10149870, Thermo Fisher Scientific), and stored at −20°C until further use.

### 4.5 Cresyl Violet and Picro Sirius Red histological staining

Cryostat sections were dried for 30 min at 37°C for adhesion to the glass slides and washed in AD. For Cresyl Violet staining, sections were immersed in Cresyl Violet solution (1% in AD, Fluka Chemicals, Sigma‐Aldrich) for 5 min and rinsed in 200 mL AD with 5 drops of acetic acid (Glacial, 100%) for 30 s. For Picro Sirius Red staining, sections were washed in 70% ethanol and AD and placed in Picro Sirius Red solution (0.1% Direct Red 80 (365548, Sigma‐Aldrich) in a saturated aquous solution of picric acid (P6477‐1GA, Sigma‐Aldrich)). Sections were washed twice in 0.5% acetic acid in AD. For both stainings, sections were then dehydrated in 100% ethanol and 100% xylol series, covered with DePeX and a coverslip, and dried overnight.

### 4.6 Senescence‐associated β‐galactosidase (SA β‐gal) assay

Sections were dried for 30 min at 37°C for adhesion and washed four times in PBS pH6. Sections were incubated in SA β‐gal solution overnight at 37°C. SA β‐gal solution was freshly prepared by adding β‐gal staining buffer (5 mM K_3_Fe(CN)_6_, 5 mM K_4_Fe(CN)6.dihydrate, 3 mM MgCl_2_ in PBS pH6) dropwise to GAL‐X (A1007, BioChemica, 1/20 in DMF) while vortexing. The next morning, sections were rinsed four times with PBS pH6 and covered with mowiol and coverslip.

### 4.7 Immunohistochemistry

Sections were dried for 30 min at 37°C for adhesion and washed in AD and TBS (0.1% Triton‐X‐100 in PBS). Heat‐mediated antigen retrieval was used to break protein cross‐links. The slices were boiled in the microwave in 1X citrate buffer (2.1 g citric acid and 500 µL Tween 20 in 1L PBS, pH 6) for 5 min at 100% and 2× 5 min at 80%. Slices were cooled down for 20 min and washed 3× 5 min with TBS. For BrdU IHC, sections were pretreated with 2N HCl at 37°C for 30 min to break the DNA and washed with 0.1 M sodium borate (in AD) to neutralize HCl. Sections were blocked for 1 h at RT with 20% normal goat serum (S26, Sigma‐Aldrich) in Tris‐NaCl blocking buffer (TNB). For IHC stainings involving the primary antibody Goat anti‐BLBP, blocking was performed with normal donkey serum (S30, Sigma‐Aldrich). Sections were stained over night with primary antibodies diluted in TNB at RT. The anti‐HuC/D antibody was incubated at 4°C for 48 h in Pierce Immunostain Enhancer (46644, Thermo Fisher Scientific). Pierce Enhancer was also used in triple IHC stainings and when the anti‐L‐plastin primary antibody was involved. The primary antibodies used were rabbit anti‐SOX2 (1:1000, SAB2701800, Sigma‐Aldrich), mouse anti‐HuC/D (1/200, A‐21271, RRID; AB_221448, Thermo Fisher Scientific), mouse anti‐pcna (1:500, ab29, RRID; AB_303394, Abcam), Goat anti‐BLBP (1:1000, ab110099, RRID; AB_10866432, Abcam), rat anti‐BrdU (1:1000, ab6326, RRID; AB_305426, Abcam), rabbit anti‐L‐plastin (1:400, GTX124420, RRID; AB_11167454, GeneTex), and mouse anti‐GS (1:1000, ab64613, RRID; AB_1140869, Abcam). Sections were washed 3× 5 min with TBS. Secondary antibodies were stained at RT in TNB for 2 h (Table S2). In case of the anti‐L‐plastin IHC staining, a long amplification was used, in which the secondary antibody is coupled to biotin (Goat anti‐Rabbit‐biotinylated, 1:300 in TNB, E043201‐8, Agilent Dako) and incubated for 45 min. After washing 3× 5 min with TBS, Streptavidin‐Cy5 (1:500 in TNB, SA1011, Thermo Fisher Scientific) was added to the sections for 2 h. For WFA staining, we used biotinylated Lectin from Wisteria Floribunda (WFA, 1:500, L1516, Sigma‐Aldrich), which was incubated overnight in TNB. The next day, Streptavidin‐Alexa 594 (1:500 in TNB, S32356, Thermo Fisher Scientific) was added for 2 h. WFA is a lectin that specifically binds *N*‐acetylgalactosamines B 1 residues of proteoglycans, including chondroitin proteoglycans (CSPGs), and has been used in neuroscience to visualize perineuronal nets (Celio & Blumcke, 1994; Härtig et al., 1992). For cell death detection, the TUNEL assay (In Situ Cell Death Detection Kit, Fluorescein, 11684795910, Sigma‐Aldrich) was used, following the manufacturer instructions. Cell nuclei were stained with 4’,6‐diamidino‐2‐fenylindool (DAPI, 1:1000 in PBS, Thermo Fisher Scientific). Sections were covered with Mowiol solution and a cover glass slide.

### 4.8 Hybridization Chain Reaction (HCR v3.0)

Probe pair generation was carried out with in situ_probe_generator (Null & Özpolat, 2020), and probe pairs (Table S3) were validated for specificity using Blastn (www.NCBI.com). HCR v3.0 was carried out as described before (Deryckere et al., 2021), which was based on the protocol of Choi and colleagues (Choi et al., 2016, 2018). The protocol was adapted to work on cryosections as follows. Cryosections were washed three times in PBS‐DEPC and once in TBS (0.3% Triton‐X‐100 in PBS). Next, sections were incubated with Proteinase K (Roche, 1:3000 in PBS‐DEPC) at 37°C for 5 min. This reaction was stopped by placing the slides in 4% PFA for 5 min. Finally, slides were washed 2 times with PBS‐DEPC and once in 5X SSCT, ready for following HCR steps (Deryckere et al., 2021).

### 4.9 Microscopy

Sections were scanned for DiD dye positivity using Texas Red light with a confocal microscope (FV1000, Olympus), to locate the injury site, even after neuroregeneration was completed. For the quantification of IHC, Cresyl Violet, and SA β‐gal stainings, a Zeiss (“Axio Imager Z1”) fluorescence microscope equipped with a AxioCam MR R3 camera (fluorescence) and a Mrc5 color camera (Bright field) was used to photograph 3 sections per animal. 20X tile scans were stitched using ZEN software (ZEN Pro 2012, Carl Zeiss). For Picro sirius red staining, polarized light was used to visualize collagen fibers using a Leica DM6 microscope and LAS X software (Leica Microsystems). For greater detail, 63X with immersion oil was applied. Channels were equally intensified for all conditions using Adobe Photoshop SC5, figure configurations were made with Adobe Illustrator (Adobe Systems).

### 4.10 Real‐time quantitative polymerase chain reaction (RT qPCR)

Young and aged killifish were euthanized in 0.1% buffered tricaine, and brains were extracted and placed on a sterile petridish. Using a 25 Gauge needle, the region surrounding the injury site was quickly punched‐out, based on DiD positivity, to ascertain that our investigations were restricted to the injury area. Tissue was rapidly snap‐frozen on dry ice, and tissue from two fish was pooled to increase sample size. RNA was extracted using the RNeasy Lipid Tissue Mini Kit (74804, Qiagen) following the manufacturer instructions. RNA was converted into cDNA using oligo dT primers and Superscript III reverse transcriptase (Invitrogen). All qPCR reactions were run in duplo with an annealing temperature of 60°C, using SYBR Green master mix (BioRad) and the CFX96/C1000 Touch Real‐Time detection system (BioRad). Gene expression values were normalized against reference genes (qBase software, Biogazelle, Table S4). Values are quantified using the comparative Ct method, with the mean of the young cycle threshold values as the control. Primer sequences (Table S4) were designed based on the *N*. *furzeri* Transcriptome browser, available on NFIN (www.NFIN.com).

### 4.11 Quantification and statistical analysis

Immunopositive cells were counted (Figure S6), and the injury area surface (mm^2^) was measured (Figure S3) on 3 sections adjacent to the lesion site and compared with corresponding sections of naive animals using, respectively, the cell counter plugin and polygon tool in Image J (Fiji). For quantification of the SA β‐gal assay, the optical density (OD) of the entire section was measured using Image J (Fiji) and divided by the OD value of the background staining (region within the section without signal). P27 signal intensity values were measured with Image J (Fiji) within a set region of interest (polygon tool) in the dorsal (D), dorso‐lateral (Dl), and dorso‐posterior (Dp) regions of the VZ. An average of these values was taken per section and divided by the signal intensity of the background. For all quantifications, an average of 3 sections was taken per animal for statistical analysis using GraphPad Prism (version 8.2.1).

Data were analyzed by 2 independent observers and first tested for Gaussian normality. If assumptions were met, we used a parametric unpaired *t* test (2 conditions) or one‐way ANOVA (> 2 conditions), followed by Dunnett's multiple comparisons test to compare injured to naive fish for each age separately. If assumptions were not met, we used the non‐parametric Mann–Whitney test (2 conditions) or Kruskal–Wallis test (> 2 conditions), followed by Dunn's multiple comparisons test. Two‐way ANOVA was used to compare young and aged fish at each time point, followed by Sidak's multiple comparisons test. n represents the number of animals in each condition. All values are mean ±standard error of the mean (SEM). Means were statistically significantly different when *p* ≤ 0.05. Only the differences between means that were found statistically significant are marked by asterixes above the bar graphs. Non‐significant differences between means are not indicated on the graphs.

## CONFLICT OF INTEREST

The authors declare that they have no conflict of interest.

## AUTHOR CONTRIBUTIONS

J.V.H. involved in conceptualization, design, experiments, statistical analysis, writing, original draft, review, editing, and visualization. V.M. and C.Z. involved in experiments, statistical analysis, review, and editing. S.V. involved in experiments. L.M., R.A., and E.S. involved in review and editing. L.A. involved in conceptualization, design, writing, review, editing, and study supervision.

## Supporting information

Fig S1

Fig S2

Fig S3

Fig S4

Fig S5

Fig S6

Fig S7

Fig S8

Table S1‐S4

Video S1

Supinfo

## Data Availability

Further information and requests for resources (killifish) should be directed to and will be fulfilled by the Lead Contact, Lutgarde Arckens (lut.arckens@kuleuven.be).
