# Supplementary figures and images for: Aging impairs the essential contributions of non‐glial progenitors to neurorepair in the dorsal telencephalon of the Killifish Nothobranchius furzeri
Source: Aging Cell. 2021 Aug 24;20(9):e13464. doi: 10.1111/acel.13464 (PMC8441397; doi:10.1111/acel.13464)

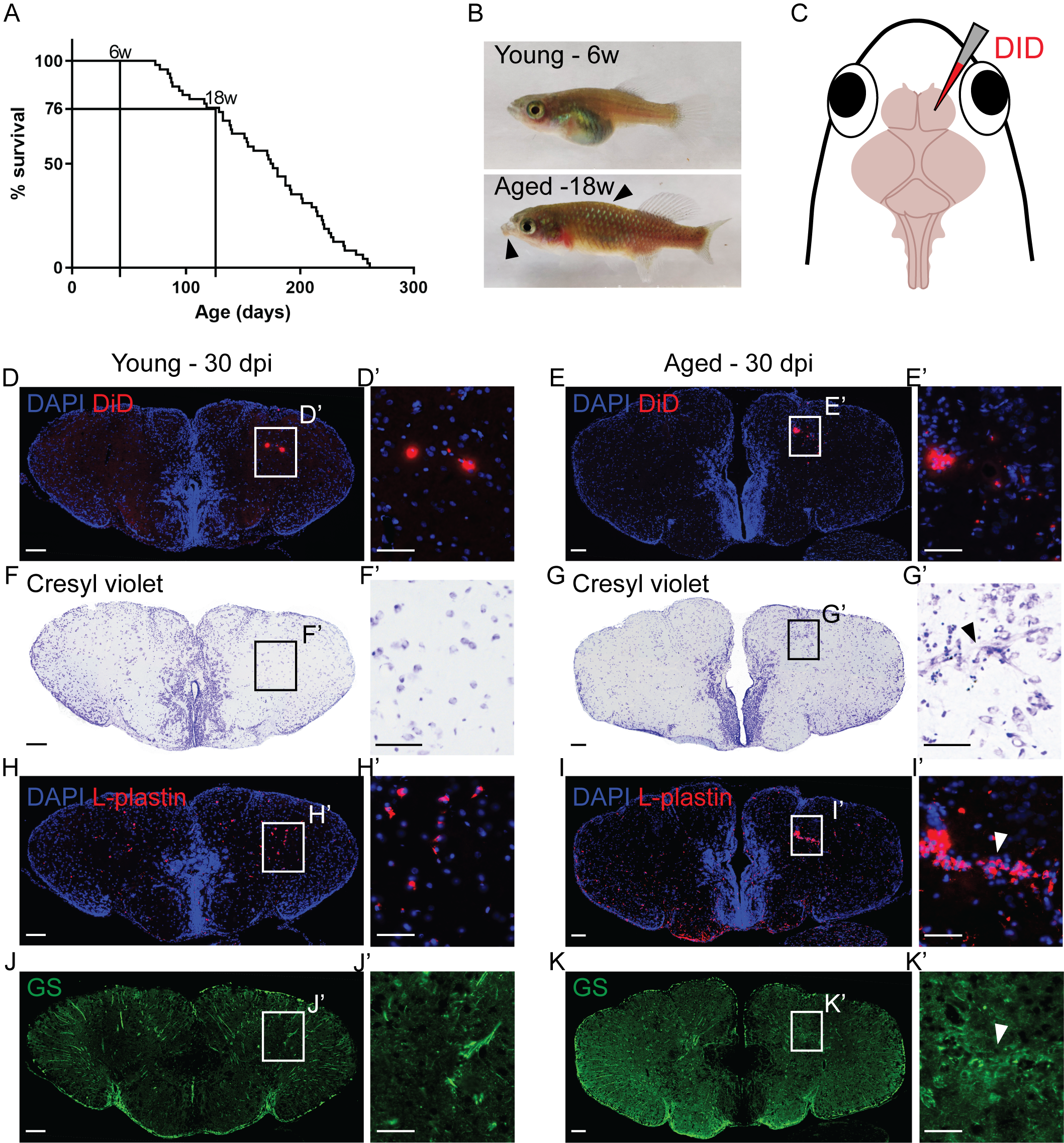

Supplement: Supplementary file 1 — Fig S1 [file ACEL-20-e13464-s003.png]

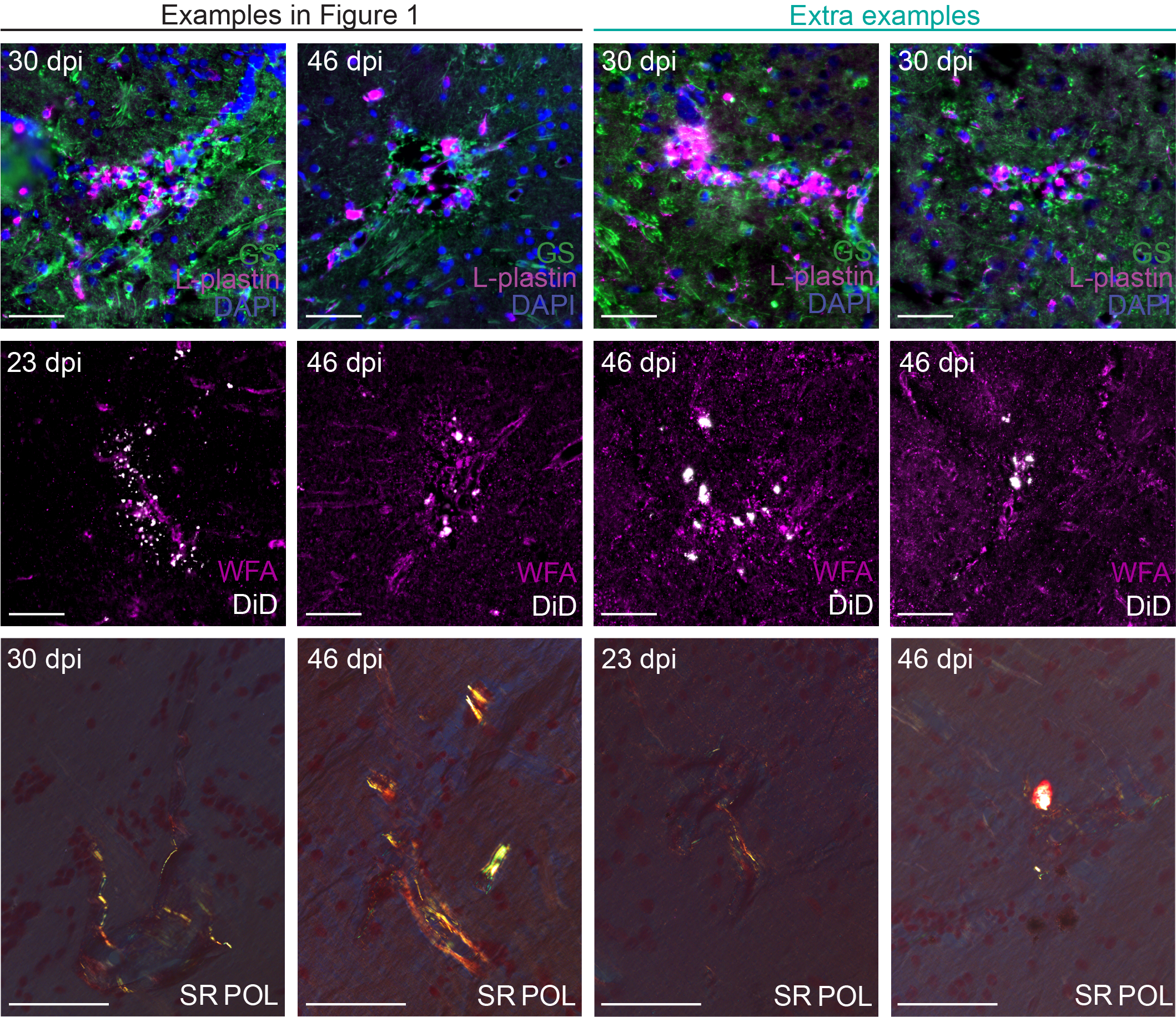

Supplement: Supplementary file 2 — Fig S2 [file ACEL-20-e13464-s005.png]

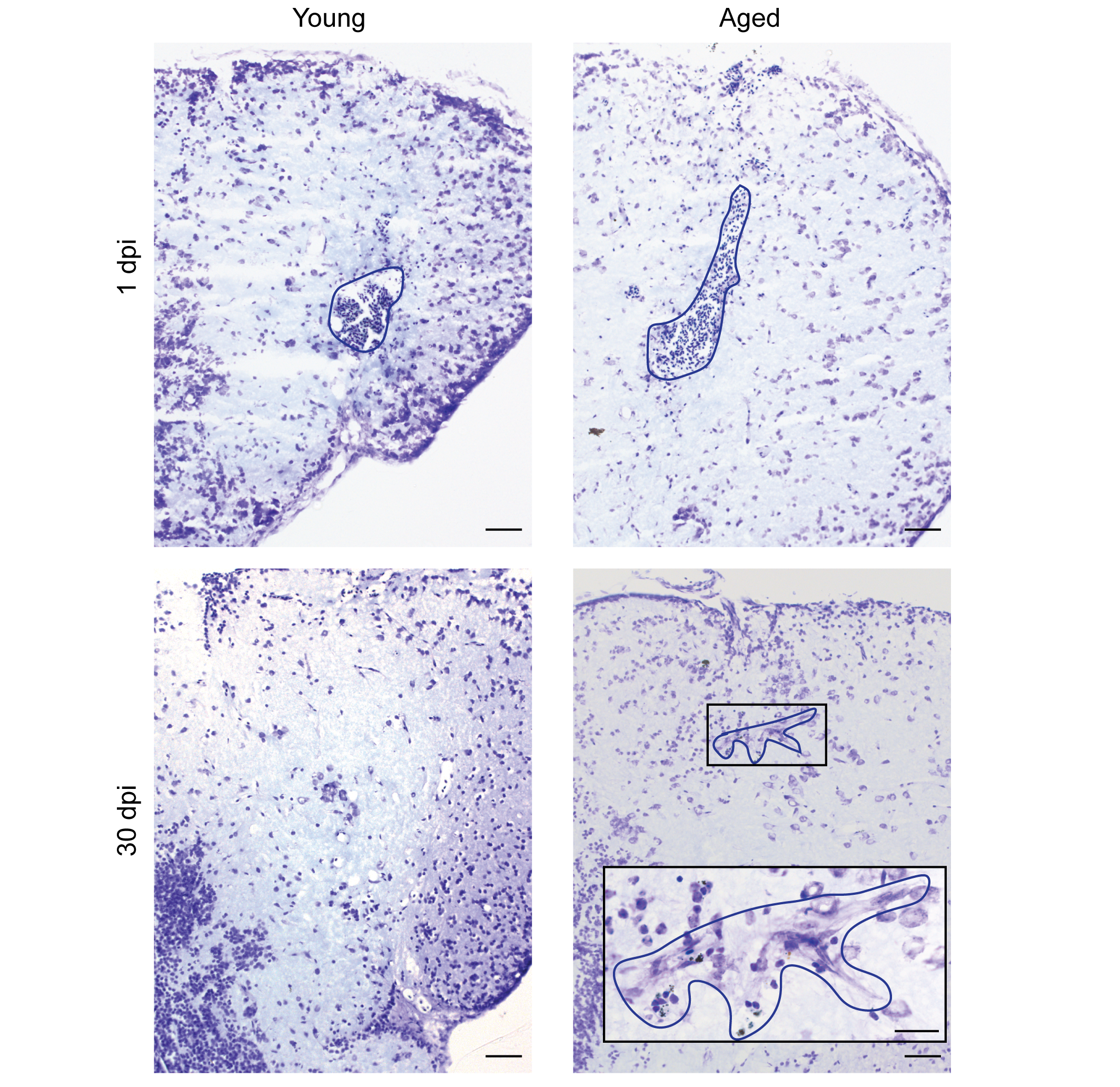

Supplement: Supplementary file 3 — Fig S3 [file ACEL-20-e13464-s011.png]

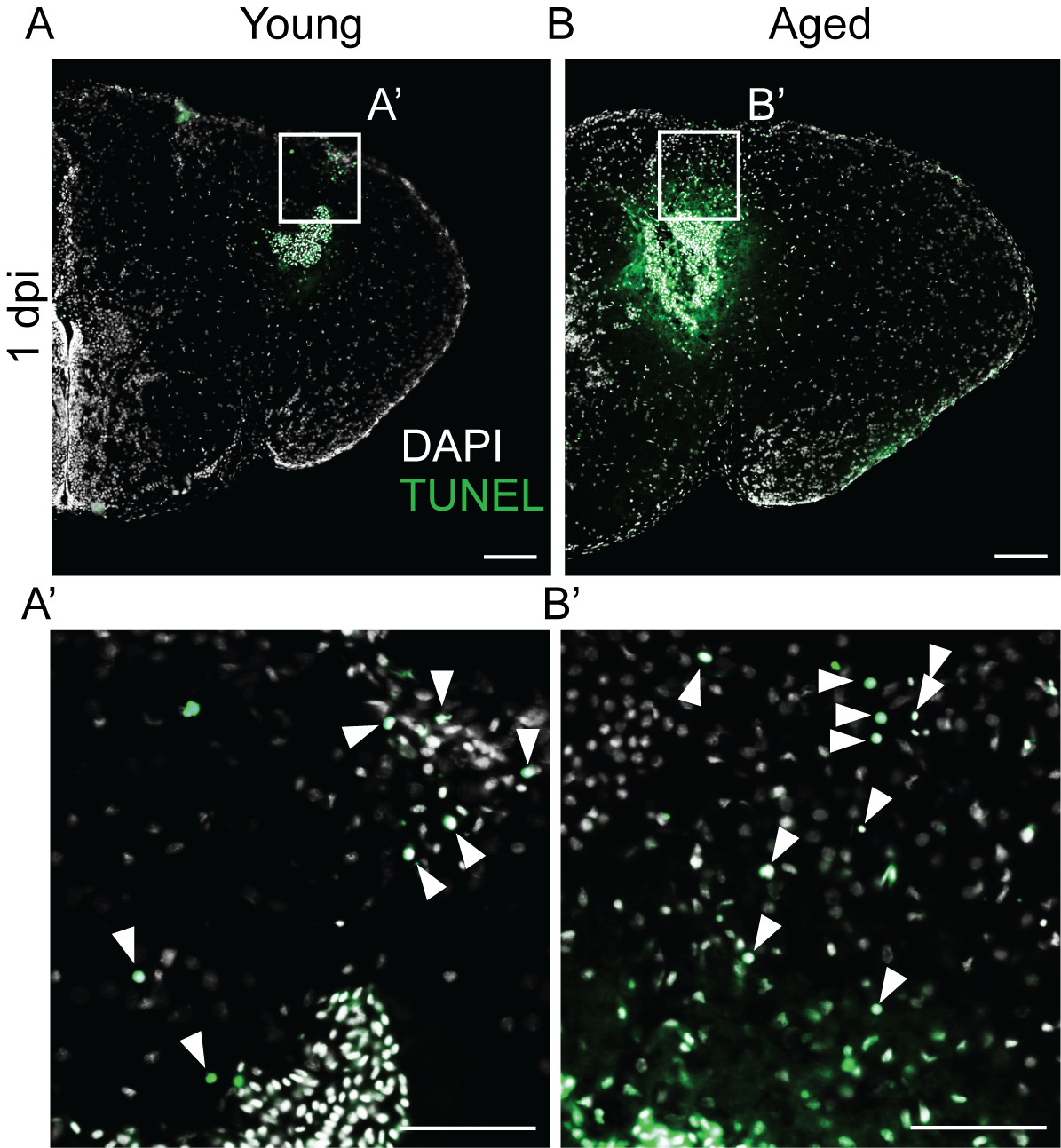

Supplement: Supplementary file 4 — Fig S4 [file ACEL-20-e13464-s008.png]

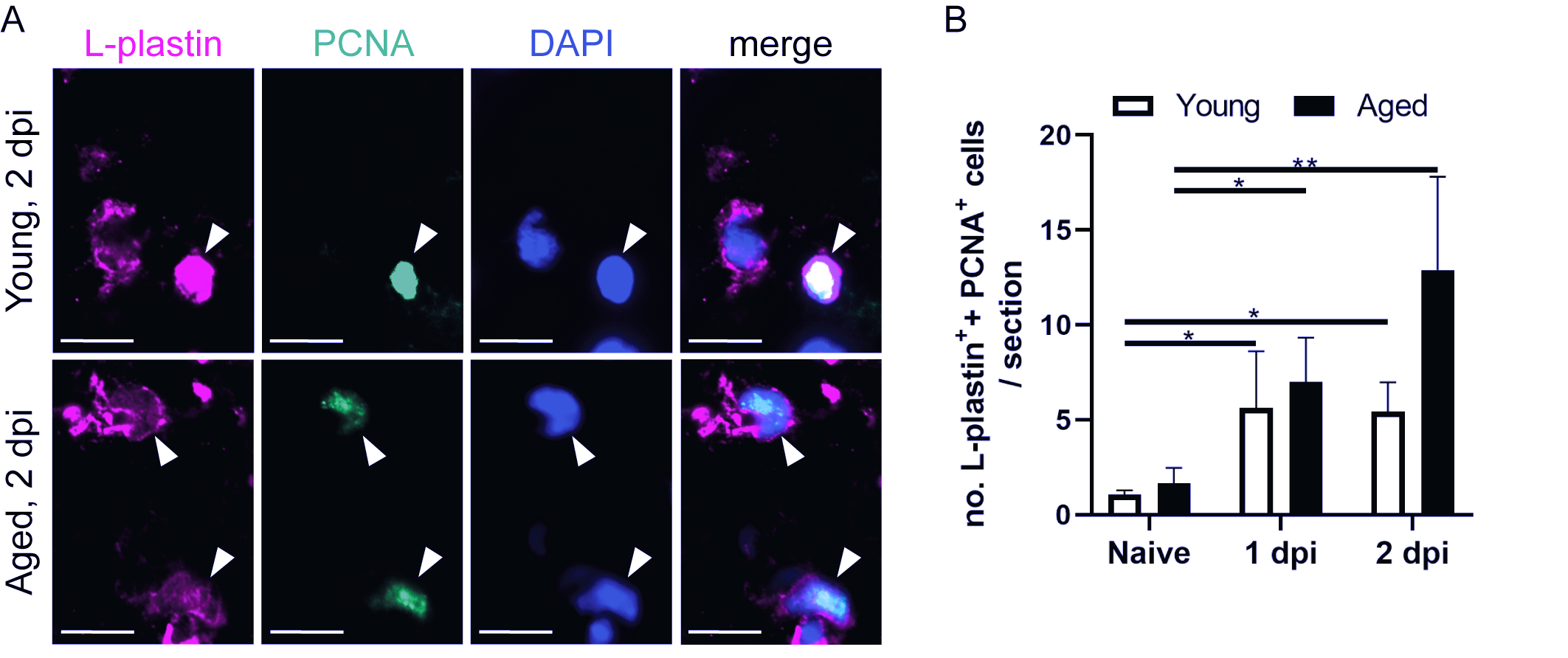

Supplement: Supplementary file 5 — Fig S5 [file ACEL-20-e13464-s001.tif]

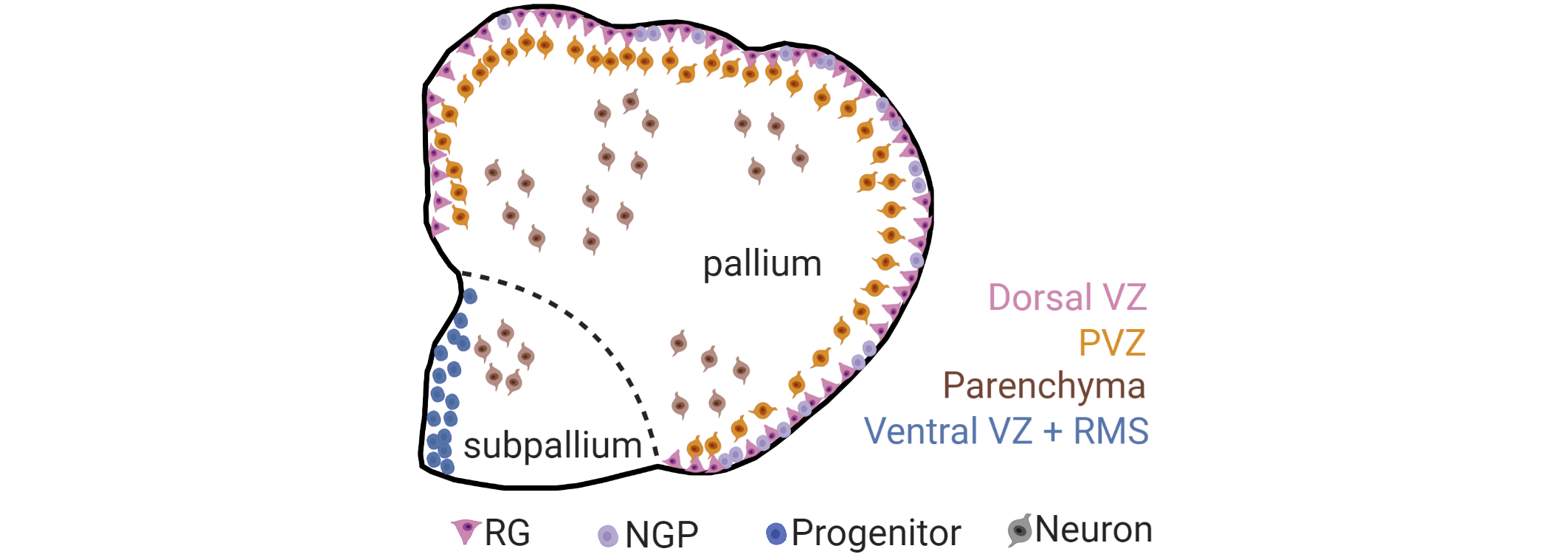

Supplement: Supplementary file 6 — Fig S6 [file ACEL-20-e13464-s007.png]

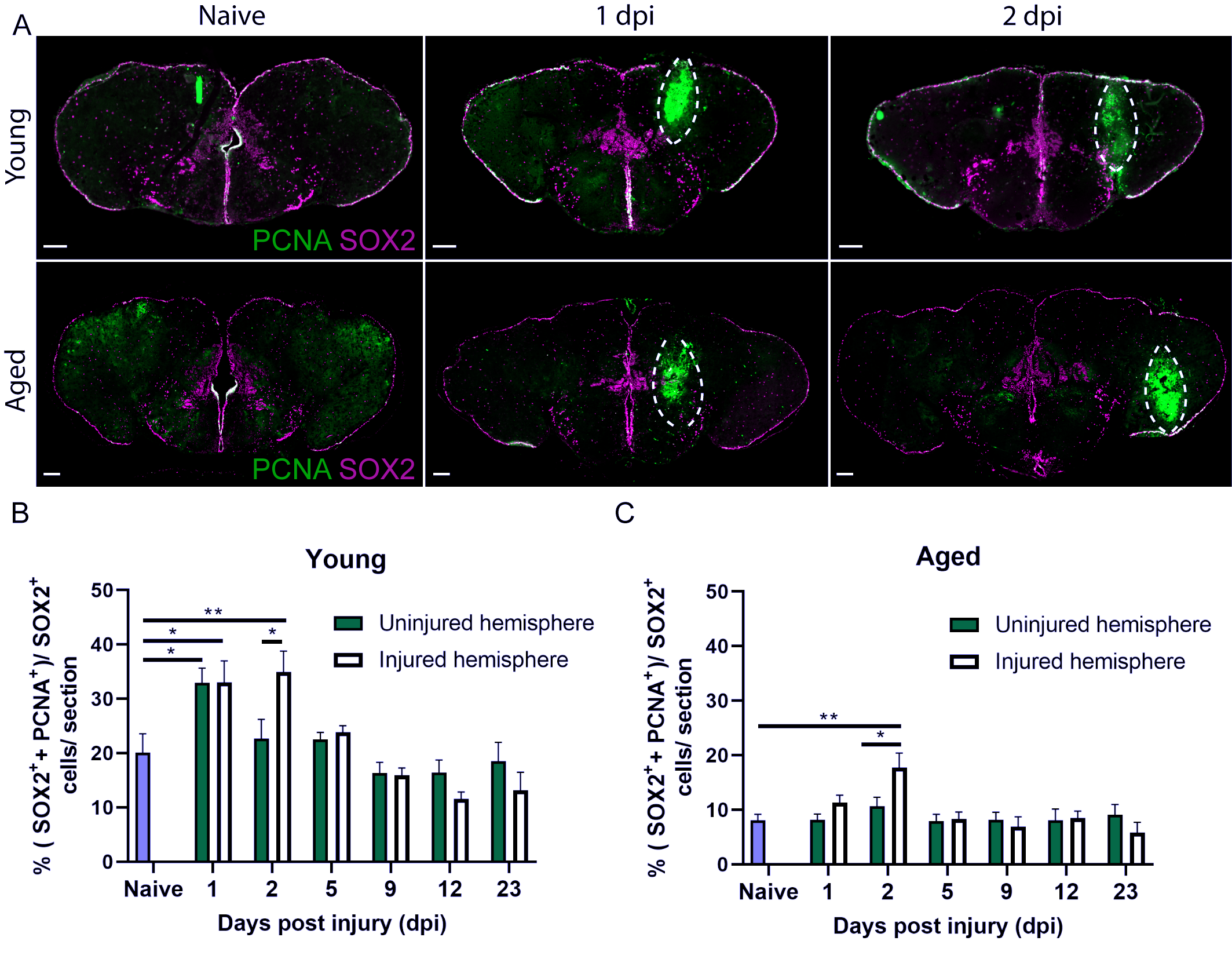

Supplement: Supplementary file 7 — Fig S7 [file ACEL-20-e13464-s009.png]

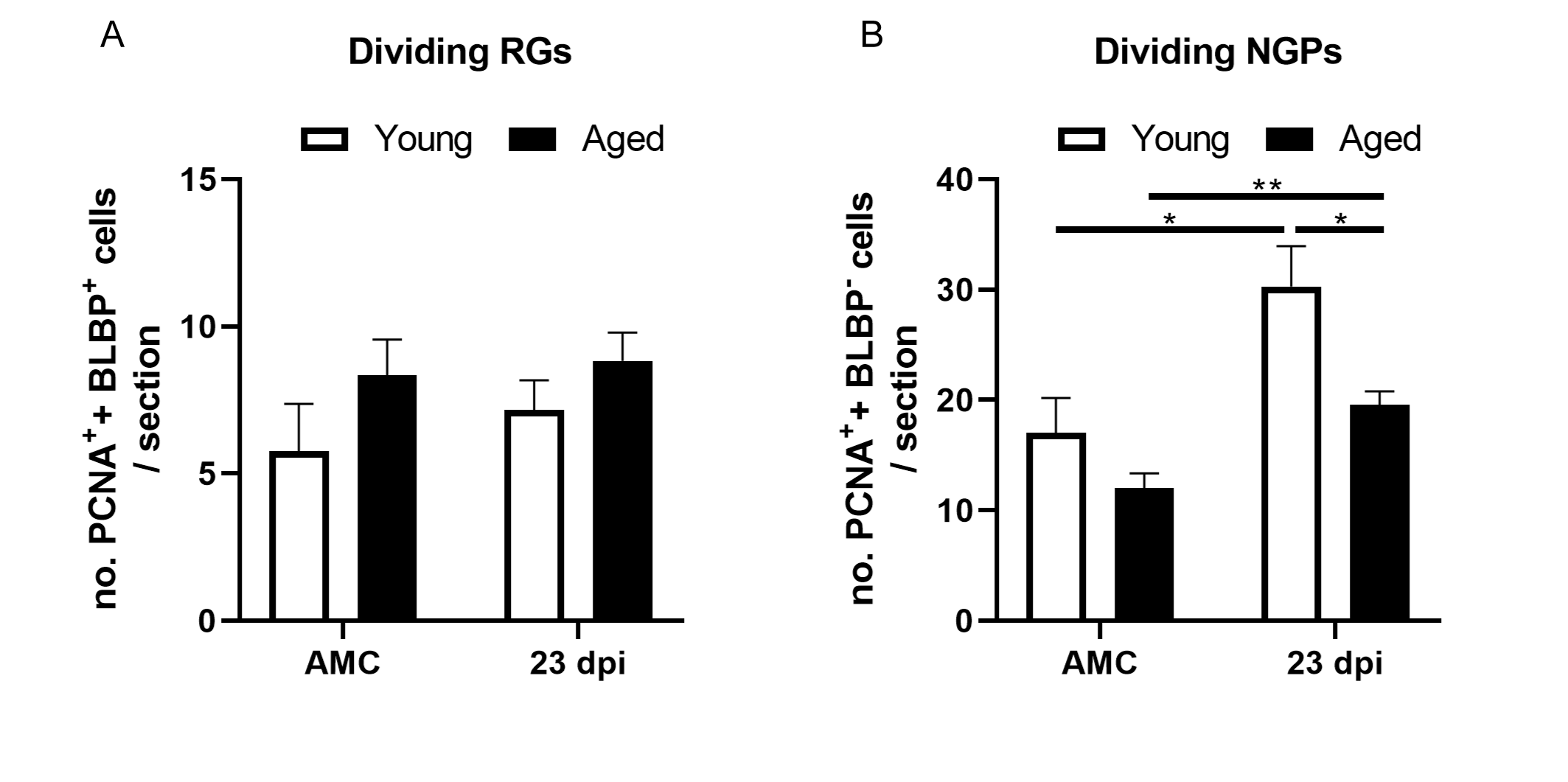

Supplement: Supplementary file 8 — Fig S8 [file ACEL-20-e13464-s010.png]
