# Supplementary material for: Aging impairs the essential contributions of non‐glial progenitors to neurorepair in the dorsal telencephalon of the Killifish Nothobranchius furzeri
Source: Aging Cell. 2021 Aug 24;20(9):e13464. doi: 10.1111/acel.13464 (PMC8441397; doi:10.1111/acel.13464)
Supplement: Supplementary file 9 — Table S1‐S4 [file ACEL-20-e13464-s002.docx]

**Supplementary table 1: Percentages of dividing RGs and NGPs among the total amount of dividing progenitors differ between young adult and aged killifish.**
Independent of injury, aged killifish display more dividing RGs in regard to the total amount of dividing progenitors compared to young adult fish. Unpaired T-test is used to compare the percentage of dividing RGs or NGPs among all dividing progenitors between young adult and aged killifish. Values are mean ± SEM; n≥5.

|  | Naive | | | 2 dpi | | |
| --- | --- | --- | --- | --- | --- | --- |
|  | Young adult | Aged | P- value | Young adult | Aged | P- value |
| % Dividing RGs | 6,7%  ±1,1 | 17,2%  ±3,2 | *P=  0,0163 | 6,5%  ±0,7 | 12,2%  ±1,8 | *P= 0,0237 |
| % Dividing NGPs | 93,3%  ±1,1 | 82,8%  ±3,2 |  | 93,4%  ±0,7 | 87,7%  ±1,8 |  |

**Supplementary table 2: Overview of secondary antibodies used for immunohistochemistry.**

| Secondary antibody | Company | Dilution |
| --- | --- | --- |
| Goat anti-mouse Alexa Fluor 488 | *Thermo Fisher Scientific Cat# A32723, RRID:AB_2633275* | 1:300 |
| Goat anti-rabbit biotinylated | *Agilent Dako Cat# E043201* | 1:300 |
| Goat anti-rabbit Alexa Fluor 594 | *Thermo Fisher Scientific Cat# A-11037, RRID:AB_2534095* | 1:300 |
| Goat anti-rat Alexa Fluor 594 | *Abcam Cat# ab150168* | 1:300 |
| Goat anti-rat Alexa Fluor 488 | *Abcam Cat# ab150165, RRID:AB_2650997* | 1:300 |
| Donkey anti-goat Alexa Fluor 594 | *Thermo Fisher Scientific Cat# A-11058, RRID:AB_2534105* | 1:300 |
| Donkey anti-goat Alexa Fluor 350 | *Thermo Fisher Scientific Cat# A-21081, RRID:AB_2535738* | 1:300 |
| Donkey anti-mouse Alexa Fluor 488 | *Thermo Fisher Scientific Cat# A-21202, RRID:AB_141607* | 1:300 |
| Donkey anti-rabbit Alexa Fluor 350 | *Thermo Fisher Scientific Cat# A10039, RRID:AB_2534015* | 1:300 |
| Donkey anti-rat Alexa Fluor 594 | *Thermo Fisher Scientific Cat# A-21209, RRID:AB_2535795* | 1:300 |

**Supplementary table 3: Nucleotide sequence of HCR probes.**

| Pool name | Nucleotide sequence |
| --- | --- |
| B2_P27 | CCTCGTAAATCCTCATCAaaCTGGTATGGGACCTCTTGTTTTGGG |
| B2_P27 | AGGTTCGGACAGCTAACCTCGTCTGaaATCATCCAGTAAACCGCC |
| B2_P27 | CCTCGTAAATCCTCATCAaaTCAGCCCGGTGCACTCCGTTTGACC |
| B2_P27 | GAGGCTCGTGACTAGCAGGTCTCTTaaATCATCCAGTAAACCGCC |
| B2_P27 | CCTCGTAAATCCTCATCAaaCCACTAGTTCCCATTTGAACCTGCC |
| B2_P27 | GGTAGAAGTCCGGCATCTCCTCGGAaaATCATCCAGTAAACCGCC |
| B2_P27 | CCTCGTAAATCCTCATCAaaTCCTTCTTTAACTCTTCGTGATCCA |
| B2_P27 | TTGAACTCCCGCATGTGTTCGTTAAaaATCATCCAGTAAACCGCC |
| B2_P27 | CCTCGTAAATCCTCATCAaaGACGCAGCTCTACACCTATGCCATG |
| B2_P27 | TATCTCCAGGTCACGGGGGAAAGATaaATCATCCAGTAAACCGCC |
| B2_P27 | CCTCGTAAATCCTCATCAaaGCGGGGCTCTGGAGTCGAAGATGGT |
| B2_P27 | ACGTCGAAGTGGAAGACGTTAACTGaaATCATCCAGTAAACCGCC |
| B2_P27 | CCTCGTAAATCCTCATCAaaCAGCACCCAATATGGCGATTGAGGA |
| B2_P27 | GCTCAGAGAGAAACTGCAGAGAAACaaATCATCCAGTAAACCGCC |
| B2_P27 | CCTCGTAAATCCTCATCAaaTTGCAACACAATTTTATCACATTAC |
| B2_P27 | TCAGATTTAATTTTCTCTTTTAATTaaATCATCCAGTAAACCGCC |
| B2_P27 | CCTCGTAAATCCTCATCAaaCAGGCTCAGTTTGCAACCTCATTGA |
| B2_P27 | AAAAAAAAAAAAACATCCAATATTTaaATCATCCAGTAAACCGCC |
| B2_P27 | CCTCGTAAATCCTCATCAaaTGTTTTTGCAAAATAGAAAAATTTT |
| B2_P27 | AGAAAAAAAAAATCCTACAAAATAAaaATCATCCAGTAAACCGCC |
| B2_P27 | CCTCGTAAATCCTCATCAaaAGAACCCACTTTTAATATCCTACAT |
| B2_P27 | AAATGCCACTTTGGATAACCGTAATaaATCATCCAGTAAACCGCC |
| B2_P27 | CCTCGTAAATCCTCATCAaaTTTCTCAGTGTTTTTTTGCATGTTT |
| B2_P27 | AGGCTTAACATAAATAAGTTAGTATaaATCATCCAGTAAACCGCC |
| B2_P27 | CCTCGTAAATCCTCATCAaaTACATTGCAAAGATTTTACACAAAT |
| B2_P27 | TTGTTTTTAACAAATTATGTACAAAaaATCATCCAGTAAACCGCC |
| B2_P27 | CCTCGTAAATCCTCATCAaaTTCTGGTGCATCCTCAGCAGGCTGA |
| B2_P27 | CAGACAGCCTGTCTGCAATGAGCATaaATCATCCAGTAAACCGCC |
| B2_P27 | CCTCGTAAATCCTCATCAaaGATCACAAAGAAGCTCCCTGTGAAG |
| B2_P27 | TGACGGGGTGAGTACAAGCATTCATaaATCATCCAGTAAACCGCC |
| B2_P27 | CCTCGTAAATCCTCATCAaaACCACGAGACTTTTTCCTTTTAGAA |
| B2_P27 | AATAAGAGGCTGGAAACTAAAAAACaaATCATCCAGTAAACCGCC |
| B2_P27 | CCTCGTAAATCCTCATCAaaATATTCCAGGACTCTGATGCTAAAA |
| B2_P27 | GAAAAACTTCAAAAAAATTGTTTCAaaATCATCCAGTAAACCGCC |
| B2_P27 | CCTCGTAAATCCTCATCAaaGGGAGGCGCTGTAATAAATGAAAAA |
| B2_P27 | TCGTTTAGACGAGTCCAGCCCTTTGaaATCATCCAGTAAACCGCC |
| B2_P27 | CCTCGTAAATCCTCATCAaaTTGTACCATTCTTCACTGCTGCATT |
| B2_P27 | AATTAAATTACGATACGTGAGCTACaaATCATCCAGTAAACCGCC |
| B2_P27 | CCTCGTAAATCCTCATCAaaGAAACGGTGAGAAGCCATGATTTTT |
| B2_P27 | AAGCTGACGGAGTGAAGGAACACTAaaATCATCCAGTAAACCGCC |
| B2_P27 | CCTCGTAAATCCTCATCAaaGCAAATGTCACACAGTCTGGAAATA |
| B2_P27 | TTTTAAGTGTAGCAATTAAGGACAGaaATCATCCAGTAAACCGCC |
| B2_P27 | CCTCGTAAATCCTCATCAaaACTGCAATTCTTCAAGTCAAATGAA |
| B2_P27 | ATAAAAAACAGCATTTTGGAAACCAaaATCATCCAGTAAACCGCC |
| B2_P27 | CCTCGTAAATCCTCATCAaaTTTAGAATTAAGTTCCTTATGTGCT |
| B2_P27 | TCACAACTTTTTACAAAAAGGATTGaaATCATCCAGTAAACCGCC |
| B2_P27 | CCTCGTAAATCCTCATCAaaATATATATCCATCTTTATTAATTTA |
| B2_P27 | CATGGAGACATATTTATACCTATATaaATCATCCAGTAAACCGCC |
| B2_P27 | CCTCGTAAATCCTCATCAaaAGCGTCCTCCTCAGTCCTCAGAAGA |
| B2_P27 | TTTAGTGTTTCTATTCAGTGCTTCAaaATCATCCAGTAAACCGCC |
| B2_P27 | CCTCGTAAATCCTCATCAaaTCATCCCCAAAAAGCCAACAAACAG |
| B2_P27 | GATATGTTTCTGATGCTGCGCAGATaaATCATCCAGTAAACCGCC |
| B2_P27 | CCTCGTAAATCCTCATCAaaGCATATAAAAAACAACACCCTCGCA |
| B2_P27 | GGCCTCCAAAAAAATAAAATAAAAGaaATCATCCAGTAAACCGCC |

**Supplementary table 4: Overview of primers used in RT-qPCR experiments.**

| Gene Name | FWD primer (5'-3') | REV primer (5'-3') |
| --- | --- | --- |
| *p27* | CGAGTTTCAAACGGGAGTC | CCCGCATGTGTTCGTTAAA |
| *p21* | GACTGCCCTGCGTAAAGAT | CAGAGGTTTGTCGGAGAAGAA |
| *Csf1ra* | CAGTTTAGTCCTCTCGTTTCC | GTGCTGGACATCTGAATCTC |
| *IL8* | ACAAATCCTGACCACAAGTAG | ATCGTATTCACCATCATGTCTC |
| Housekeeping genes | | |
| *EF1a* | ACTCTGGCATTGTCGTTTAG | AGTTACCAGCAGCTTTCTTC |
| *Tuba* | CAGATGGTCAAGTGTGATCC | AGTTGGAGGCTGGTAGTT |
| *TBP* | CTCACAGTTACAGGACCAAAC | TCCGAACCTCAAAGAGAAGA |

**Supplementary movie 1: Detailed description of the stab-wound injury model used in this study.**
