# Supplementary material for: Aging impairs the essential contributions of non‐glial progenitors to neurorepair in the dorsal telencephalon of the Killifish Nothobranchius furzeri
Source: Aging Cell. 2021 Aug 24;20(9):e13464. doi: 10.1111/acel.13464 (PMC8441397; doi:10.1111/acel.13464)
Supplement: Supplementary file 11 — Supinfo [file ACEL-20-e13464-s006.docx]

# Supplementary figure legends

**Supplementary figure 1: Research methodology**

**(A)** Survival curve of *N. furzeri* females (strain GRZ-AD), housed in a Tecniplast ZebTEC multi-linking aquarium system (n=48). Six week old killifish are chosen as young adult based on 100% survival rate and having reached sexual maturity. Eighteen week old killifish are chosen as aged based on 76,6% survival rate and showing phenotypic aging hallmarks (illustrated in B). **(B)** Photographs of a young adult (6 week old) and aged (18 week old) *N. furzeri* female (strain GRZ-AD). Aged females show phenotypic aging hallmarks, such as a spinal curvature and protrusion of the lower lip (arrowheads). **(C)** Schematic of the stab-wound injury method. After removing skin above the skull, a 33-gauge Hamiliton needle is pushed into the medial zone of the right telencephalic hemisphere. The needle is dipped in DiD solution (red), which labels the membranes of cells around the injury site (illustrated in D,E). **(D-K)** The injury site can be found by scanning sections for DiD-positivity (red crystals) after cryosectioning, for example at 30 dpi. **(D,F,H, J)** Adjacent coronal sections of a young adult killifish telencephalon. (D) The DiD dye is still visible at 30 dpi. (F) Cresyl violet staining shows that the tissue of the young adult fish is structurally regenerated. (H) No glial scar (or malformation) is visible after staining for L-plastin^+^ microglia/macrophages in the young adult brain at the site of injury. (J) GS^+^ RG fiber distribution is normal in young adult fish at 30 dpi. **(E,G,I,K)** Adjacent coronal sections of an aged killifish telencephalon. (E) The DiD dye clearly marks the injury site at 30 dpi. (G) Cresyl violet staining shows tissue scarring (arrowhead in G’), indicative for incomplete repair. (I) Signs of glial scarring are visible after staining for L-plastin^+^ microglia/macrophages in the aged brain at the site of injury. (K) GS^+^ RG fibers are in close contact with the glial scar (arrowhead in K’). Scale bars in D-I: 100 µm. Scale bars in D’-I’: 50 µm. dpi: days post injury.

**Supplementary figure 2: Representation of the interindividual variability in glial scarring in aged killifish.** The glial component of the scar is best visualized by GS and L-plastin double stainings. The glial scar is always defined by a cluster of L-plastin^+^ microglia/macrophages, surrounded by GS^+^ RG fibers, yet the size of the cluster varies between fish. The extracellular matrix component of the glial scar is best visualized with a WFA staining and/or a Picro sirius red staining, showing deposition of proteoglycans and collagen respectively at the site of injury (DiD-positivity). The amount of deposition varies between animals but the two features are detected in every animal. Scale bars: 50 µm. dpi: days post injury, SR: Picro sirius red, POL: polarized light, WFA: Wisteria Floribunda agglutinin, GS: glutamine synthetase, RG: radial glia. GS and L-plastin staining: the third panel from the left is a merge of panels I’,K’ of Figure S1.

**Supplementary figure 3: Methodology of injury surface area measurements**

In ImageJ (FIJI) the scale is set and a polygon is drawn around the injury site with the polygon tool (blue line). ImageJ calculates the surface of the polygon. At early stages, the injury site is visible by a blood clot and the borders of this clot are taken as the injury borders (young adult, aged at 1 dpi, scale bar: 50 µm). In later stages, malformation of the parenchymal tissue is visible (aged 30 dpi, scale bar: 50 µm). In this case, the borders of this malformation are taken as the injury borders (illustrated in the inset of aged 30 dpi, scale bar: 20 µm).

**Supplementary figure 4: Aged killifish have higher numbers of apoptotic cells at 1 dpi**

TUNEL assay reveals TUNEL^+^ apoptotic cells (green) overlapping with DAPI (white) in young adult **(A)** and aged **(B)** killifish at 1 dpi. Aged killifish have more TUNEL^+^ cells compared to young adult killifish. Scale bars in A, B: 100 µm. Scale bars in A’, B’: 50 µm.

**Supplementary figure 5: Injury induces proliferation of microglia in young adult and aged fish**

**(A)** Double staining for L-plastin (magenta) and PCNA (green) with DAPI (blue) shows proliferating microglia/macrophages (arrowhead) on coronal sections of young and aged killifish at 2 dpi. Scale bar: 10 µm. **(B)** Absolute number of L-plastin^+^ PCNA^+^ proliferating microglia/macrophages in young adult and aged telencephali in naive conditions and at 1 and 2 dpi. Microglia/macrophages start proliferating early after injury in the killifish telencephalon. Significantly higher levels of proliferating microglia/macrophages are observed at 1 and 2 dpi for both ages. This activation is slightly more pronounced but highly variable in aged killifish compared to young adult fish. *p≤0,05, **p≤0,01; One-way ANOVA is used to compare naive fish to injured fish for each. Young: parametric one-way ANOVA, followed by Dunnett’s multiple comparisons test. Aged: non-parametric Kruskal-Wallis test, followed by Dunn’s multiple comparisons test. Two-way ANOVA is used to compare young and aged fish, followed by Sidak's multiple comparisons test. Values are mean ± SEM; n≥5.

**Supplementary figure 6: Counting method**

Schematic view of the different regions in which different types of cells are counted in the right telencephalic hemisphere. VZ: ventricular zone, PVZ: periventricular zone, RMS: rostral migratory stream, RG: radial glia, NGP: non-glial progenitor. Created with BioRender.com.

**Supplementary figure 7: Injury only acutely increases neural progenitor cell proliferation in the uninjured hemisphere in young killifish but not in aged killifish**

**(A)** Double staining for SOX2 (magenta) and PCNA (green) on coronal sections of young and aged killifish in naive conditions and at 1 and 2 dpi. The dashed lines encircle the site of injury filled with blood cells that autofluoresce in the green channel. Scale bars: in A: 100 µm. **(B,C)** Proportion of double positive SOX2^+^ PCNA^+^ dividing progenitor cells among SOX2^+^ cells (all progenitor cells) in young adult (B) and aged (C) uninjured and injured hemispheres at 1, 2, 5, 9, 12 and 23 dpi and in naive animals. (B) At 1 dpi, the uninjured hemisphere of young adult fish contains a higher percentage of dividing progenitors, compared to naive animals, suggesting systemic effects of the stab-wound injury on the uninjured hemisphere. (C) The uninjured hemisphere contains similar percentages of dividing progenitors as naive animals for all time points, suggesting that no systemic effects are at play in aged killifish. *p≤0,05, **p≤0,01; One-way ANOVA is used to compare naive fish to the uninjured hemisphere, followed by Dunnett's multiple comparisons test. Two-way ANOVA is used to compare the uninjured and injured hemisphere at each time point, followed by Sidak's multiple comparisons test. Values are mean ± SEM; n≥5, except for aged, 9 dpi: n=4. dpi: days post injury.

**Supplementary figure 8: The dividing progenitor pool is not depleted within a short period after injury**

Absolute number of BLBP^+^ PCNA^+^ dividing RGs and **(B)** BLBP^-^ PCNA^+^ dividing NGPs in the VZ of young and aged telencephali in AMC and at 23 dpi. Even after the production of neurons, the number of dividing RGs and NGPs is respectively similar or increased at 23 dpi compared to AMCs. This suggests that killifish replenish the proliferative stem cell pool after injury. *p≤0,05, **p≤0,01, Unpaired t-test or non-parametric Mann Whitney test is used to compare AMC to 23 dpi fish. Two-way ANOVA is used to compare young and aged fish, followed by Sidak's multiple comparisons test. Values are mean ± SEM; n≥5. RG: radial glia, NGP: non-glial progenitor, AMC: age-matched control, VZ: ventricular zone, dpi: days post injury.
